# Structure-Based Identification of Non-covalent Prolyl Oligopeptidase 80 Inhibitors Targeting *Trypanosoma cruzi* Cell Entry

**DOI:** 10.1021/acs.jcim.4c02152

**Published:** 2025-02-26

**Authors:** Vinícius Alexandre Fiaia Costa, Flávia Nader Motta, Alexandra Maria dos Santos Carvalho, Felipe da Silva Mendonça de Melo, Melina Mottin, Sébastien Charneau, Philippe Grellier, Jaime Martins Santana, Izabela Marques Dourado Bastos, Bruno Junior Neves

**Affiliations:** † Laboratory of Cheminformatics, Faculty of Pharmacy, Federal University of Goiás, Goiânia 74605-170, Brazil; ‡ Pathogen-Host Interface Laboratory, Department of Cell Biology, Institute of Biological Sciences, University of Brasilia, Brasilia 70910-900, Brazil; § Laboratory of Biochemistry and Protein Chemistry, Department of Cell Biology, Institute of Biological Sciences, University of Brasilia, Brasilia 70910-900, Brazil; ∥ UMR, Molécules de Communication et Adaptation des Micro-organismes, Muséum National d’Histoire Naturelle, Équipe Parasites et Protistes Libres, 7245 Paris, France

## Abstract

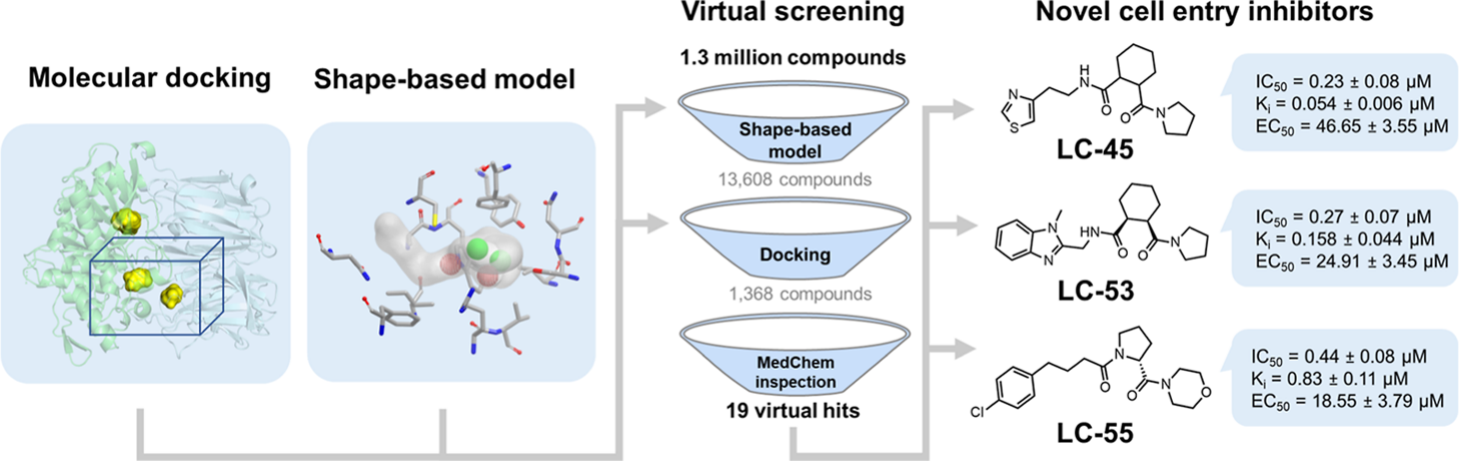

Chagas disease remains a persistent public health challenge due to the limited efficacy and significant toxicity of current pharmacological treatments. This highlights the urgent need for novel drugs with innovative mechanisms of action, specifically targeting cell infection pathways. The prolyl oligopeptidase of *Trypanosoma cruzi* (POPTc80) has emerged as a promising target for developing inhibitors to block the parasite’s infection process. In this study, we developed a robust structure-based virtual screening pipeline to discover potent POPTc80 inhibitors. The customized protocol integrated structural analysis of the 3D structure of POPTc80 and enrichment analysis of molecular docking and shape-based models to optimize the selection of potential inhibitors. After optimization, a large-scale virtual screening of 1.3 million compounds prioritized 19 putative hits for experimental validation. Nine of these compounds demonstrated inhibitory activity at nanomolar concentrations. The most potent inhibitors—LC-44 (*K*_*i*_ = 0.175 μM), LC-45 (*K*_*i*_ = 0.054 μM), LC-46 (*K*_*i*_ = 0.513 μM), LC-50 (*K*_*i*_ = 0.44 μM), LC-53 (*K*_*i*_ = 0.158 μM), and LC-55 (*K*_*i*_ = 0.83 μM)—demonstrated superior inhibitory activity, consistent with the competitive inhibition mechanism predicted by our computational protocol. Subsequently, a phenotypic assay confirmed their ability to effectively inhibit *T. cruzi* entry into host cells in a dose-dependent manner, further validating their mechanism of action. These findings establish these compounds as promising chemical scaffolds for prospective hit-to-lead optimization, offering a unique opportunity to develop novel, mechanism-driven therapeutics targeting a critical step in the parasite’s infection process.

## 1 Introduction

Chagas disease is a potentially chronic, life-threatening condition caused by the protozoan parasite *Trypanosoma cruzi*. It is one of the most prevalent neglected tropical diseases associated with poverty. The World Health Organization estimates that 6 to 7 million people in 21 Latin American countries are infected with *T. cruzi*, resulting in 12,000 deaths annually.^1−3^ However, its distribution has changed recently due to climate change and increased migratory movements reaching North America and Europe.^4,5^

Parasite transmission to humans occurs through the feces and urine of infected Triatominae bugs during the blood meal. Other transmission routes include oral ingestion of fresh food contaminated with infectious forms of the parasite, blood transfusion, transplacental passage from mother to fetus, and organ transplantation. The disease manifests in two phases: an acute phase characterized by high parasitemia and nonspecific symptoms and a chronic phase that can lead to severe cardiac,^6^ gastrointestinal,^7^ and neurological^8^ complications.

Current treatment relies on two drugs developed over five decades ago: benznidazole and nifurtimox. These nitroheterocyclic drugs require long treatment durations and have significant drawbacks, including toxicity, lengthy regimens, the emergence of drug-resistant *T. cruzi* strains, and variable efficacy (ranging from 2 to 40%), particularly in the chronic phase.^9−11^ These limitations underscore the need for new drugs with innovative mechanisms of action.

An innovative strategy to tackle Chagas disease involves targeting the mechanisms of *T. cruzi* host cell invasion, offering a novel approach to drug discovery.^12^ In this context, the 80 kDa prolyl oligopeptidase of *T. cruzi* (POPTc80) emerged as a promising target for developing cell entry inhibitors. POPTc80 is a serine protease crucial for the parasite’s invasion capabilities. It is expressed in the infective trypomastigote and the replicative intracellular amastigote forms of the parasite.^13^ Unlike other POPs, POPTc80 can hydrolyze large substrates such as fibronectin and collagen type I and IV, which are essential components of the extracellular matrix.^14^ This enzymatic activity facilitates the parasite’s migration through host tissues and entry into nonphagocytic cells. Furthermore, selective inhibitors of POPTc80 have been developed and shown to block parasite infection in vitro, demonstrating the enzyme’s potential as a viable target for drug development efforts to inhibit parasite invasion and propagation.^13,15−19^ Given its critical role in *T. cruzi* infection, targeting POPTc80 represents a promising strategy for developing effective anti-Chagas therapies.

To address this need, computer-aided drug design (CADD) techniques provide a powerful approach to identifying novel POPTc80 inhibitors. Among them, molecular docking tools are well-placed in modern drug discovery pipelines to model the interaction between small molecules and proteins at the atomic level.^20−22^ This allows researchers to screen large chemical libraries and prioritize putative hits for experimental validation. However, the effectiveness of molecular docking is constrained by the low accuracy of scoring functions^21−25^ and a reduced sampling of macromolecule conformations in pose prediction.^26,27^ Both limitations negatively impact the hit rates in virtual screening (VS) campaigns, leading to the random prioritization of inactives for experimental validation.

In this study, we developed a comprehensive structure-based virtual screening pipeline incorporating advanced computational analyses and validation protocols to prioritize novel POPTc80 inhibitors targeting *T. cruzi* cell entry. The screening efforts identified 19 putative inhibitors, which were subsequently subjected to in vitro enzymatic assays, ultimately leading to the discovery of three potent inhibitors with nanomolar activity against POPTc80. Furthermore, a phenotypic assay confirmed that these inhibitors effectively inhibit *T. cruzi* cell entry in a dose-dependent manner, reinforcing their potential as promising hits for prospective hit-to-lead investigations.

## 2 Methods

### 2.1 Computational

#### 2.1.1 3D Structure Prediction and Refinement

The FASTA sequence of POPTc80 (accession code: TcCLB.506247.230) was retrieved from TriTrypDB^28^ and used as input for 3D structure prediction via three approaches: (i) homology modeling implemented in SWISS-MODEL server,^29^ (ii) threading method available on I-TASSER server,^30^ and (iii) *ab initio* modeling based on graph attention neural network available on AlphaFold server.^31 ,32^ Then, 3D POPTc80 models were structurally refined in the GalaxyRefine server,^33,34^ which performs iterative optimization with global geometric operators (e.g., anisotropic normal mode perturbation and secondary structure perturbation) and local operators (e.g., loop modeling and hybridization) to improve the accuracy of the initial model.

##### 2.1.1.1 Geometric Analysis

The overall geometrical quality of refined models was investigated through the MolProbity server.^35,36^ Models with at least 98% Ramachandran angles (phi [ϕ] and psi [ψ]) in favored regions, along with the lowest Clashscore and MolProbity scores were selected for further analysis. The Clashscore represents the number of serious steric clashes per 1000 atoms. The MolProbity score is a log-weighted combination of the percentage of bad side-chain rotamers, the percentage of Ramachandran outliers, and Clashscore, giving one number that reflects the resolution of the experimentally solved structures at which those values would be expected.

#### 2.1.2 Normal Mode Analysis

The POPTc80 model was imported into the DynOmics server^37^ to generate representative conformations for subsequent molecular modeling simulations. The DynOmics integrates two widely used elastic network models (ENSs): (i) the Gaussian network model (GNM)^38^ and (ii) the anisotropic network model (ANM).^39^ GNM was used to sample the conformational dynamics of POPTc80 through analysis of contact topology in a coarse-grained presentation in the environment.^38^ The ANM uses an elastic network (EN) to explore vibrational motions in the molecular system through graphs. Here, each POPTc80 node represents a Cα atom of an amino acid residue and the overall potential is simply the sum of harmonic potentials between interacting nodes.^39^

#### 2.1.3 Binding Site Identification

A Principal Component Analysis (PCA) with k-means implemented in the Bio3D package^40^ was performed on the generated POPTc80 conformations to capture significant conformational changes and their interconformer relationships. From this analysis, the most representative conformations were identified and further refined using the FTSite server.^41^ FTSite detects potential binding pockets by mapping interactions with 16 diverse small organic molecules systematically arranged around the POPTc80 structure.^41^ Special attention was given to selecting conformations with significant conformational differences, especially those indicating changes in pocket geometry, potentially revealing cryptic sites. This approach ensured a comprehensive characterization of key binding sites and hotspots.

#### 2.1.4 Analysis of Evolutionary Conservation Profile

The prioritized POPTc80 conformations were submitted to the ConSurf server^42^ to estimate the evolutionary conservation of amino acids based on phylogenetic relationships with homologues. Initially, 150 homologous sequences were imported from the UNIREF-90 database^43^ using an *E*-value cutoff of 10^–5^. Redundant sequences (identity >95%) or sequences with minimal identity (<35%) with the POPTc80 were ignored.^42^ Then, a multiple sequence alignment (MSA) of homologous sequences was built using the MAFFT-L–INS–i method,^44^ whereas a phylogenetic tree was built using the neighbor-joining algorithm.^45^ Finally, the evolutionary conservation scores of position-specific amino acids were computed using the empirical Bayesian method.^46^

#### 2.1.5 Protein Preparation

The selected POPTc80 conformations were imported into Maestro workspace v.9.3 (Schrödinger, LCC, New York, 2012) and processed with the Protein Preparation Wizard. In this step, hydrogen atoms were added to the proteins, while bond orders and formal charges were adjusted. Further, the Epik program^47^ was employed to predict the protonation states (p*K*_a_) of polar amino acids at pH = 7.4 ± 0.5, whereas the PROPKA v.3.1^48,49^ was used to optimize the hydrogen’s orientations.

#### 2.1.6 Building a Benchmark Data Set

We retrieved a series of 24 POPTc80 inhibitors with IC_50_ or *K*_*i*_ ≤ 10 μM from the literature.^15−19^ Each compound in the series was structurally standardized according to the protocol proposed by Fourches and coauthors.^50−52^ Specific chemotypes, such as aromatic and nitro groups, were normalized. In addition, polymers, salts, organometallic compounds, and mixtures were also removed. Then, 864 decoys were selected from the ZINC drug-like subset.^53^ Decoys were chosen if they were similar to active compounds according to five physicochemical descriptors (cLogP, number of rotational bonds, number of hydrogen bond donors and acceptors, and molecular weight) and structurally different from the active compounds (Tanimoto coefficient ≤0.85). The low-energy conformers and protonation states for both actives and decoys were predicted using LigPrep 2.5 at a pH of 7.4 ± 0.5.

#### 2.1.7 Molecular Docking Protocol

Six grid boxes were established into *x*, *y*, and *z* coordinates of POPTc80 using the receptor grid generation panel of Glide v.5.8.^54^ Details of grid boxes are shown in Supporting Information Table S1. They were generated to assess the most suitable combinations of the binding site and conformation for the docking, thus improving enrichment rates in VS. Then, molecular docking calculations were performed on the Maestro workspace Glide v.5.8,^54,55^ employing high-throughput virtual screening (HTVS), standard precision (SP), and extra precision (XP) modes. The poses were scored using GlideScore, Glide Energy, and Glide Emodel functions^54,55^ and further optimized using the OPLS2005 force field,^56^ which ensures that the poses are refined locally.

#### 2.1.8 Shape-Based Modeling

The docking poses of six known POPTc80 inhibitors (see Table S2) were loaded into the ROCS program v.3.2.2.2^57,58^ and used as molecular queries for developing shape-based models. The intermolecular interactions of queries with the POPTc80 binding site were used as criteria to rationalize the color features of shape-based models. Then, 500 conformers were generated for each compound of the benchmark data set using OMEGA v.3.0.0.1 software,^59,60^ while the AM1-BCC charges^61^ were added using QUACPAC v.1.7.0.2.^62^ The output conformations of compounds were aligned by a solid-body optimization process that maximizes the overlap volume with queries and ranked according to the RefTverskyCombo scoring function.^63^ RefTverskyCombo measures molecular similarity or dissimilarity using two basic components: (i) the representation of molecular characteristics (such as shape and color) and (ii) the Tversky similarity coefficient that is used to quantify the degree of resemblance between two such representations.^57,58^

#### 2.1.9 Enrichment Analysis

The enrichment rates of the docking and shape-based models were calculated using Area Under the ROC Curve (AUC), Enrichment Factor (EF), and Boltzmann-Enhanced Discrimination of ROC (BEDROC).^21^ The AUC and EF were calculated as follows
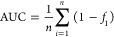
1
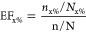
2where *f*_1_ is the fraction of decoys ranked higher than the *i*-th active, *n* represents the total number of actives, *N* represents the total number of compounds (actives and decoys) in the benchmarking data set, whereas *n*_*x*%_ and *N*_*x*%_ represents the number of actives and represents the total number of compounds in the *x*% ordered list, respectively. Although extremely important, the AUC and EF cannot discern the order of actives and decoys in the top *x*% list.^21^ To overpower the “early recognition” issue, BEDROC was calculated as follows
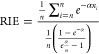
3
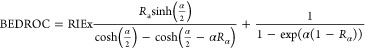
4where *x*_*i*_ is the relative rank of the *i*-th active, α is an exponential weighting factor that controls the emphasis given to early recognition, and *R*_α_ is the proportion of actives in the benchmarking set.

#### 2.1.10 Virtual Screening

The best shape-based model and docking protocols were employed as filters for VS of ∼1.3 million compounds from the CORE and EXPRESS-Pick collections of the ChemBridge database (http://www.chembridge.com/) aiming to identify new cell entry inhibitors with activity against POPTc80. Finally, the selected virtual hits were purchased and validated using in vitro enzymatic assays.

### 2.2 Experimental Section

#### 2.2.1 Materials

Test compounds were sourced from the ChemBridge database located in San Diego, CA, USA. These compounds were then resuspended in 100% dimethyl sulfoxide (DMSO) to create a stock solution at a concentration of 40 mM. The solution was aliquoted and subsequently stored at −80 °C. All chemical structures were confirmed using proton (^1^H) NMR spectra MHz or liquid chromatography–mass spectrometry (LC–MS) analysis with evaporative light scattering and ultraviolet detectors confirmed a minimum purity of 95% for all compounds (spectra of compounds are listed in Supporting Information). LIT media was purchased from Vitrocell Embriolife (Campinas-SP, Brazil). All other reagents were purchased from Sigma-Aldrich (St. Louis-MO, USA).

#### 2.2.2 Enzyme Inhibition

The enzyme activity was assessed using the fluorogenic substrate Suc-GPLGP-AMC previously described by Santana et al.^14^ A total of 20 ng of pure recombinant POPTc80 was incubated with 20 μM Suc-GPLGP-AMC in a 25 mM HEPES buffer containing 5 mM DTT at pH 7.5. The release of AMC was monitored over a period of 20 min by measuring its emission at 460 nm, following excitation at 355 nm, using a 96-well microplate fluorescence reader (M5, Molecular Devices). The IC_50_ values were obtained by a series of at least 9 points from 200 to 0.01 μM of the compound, plus the control. The data were subjected to a four-parameter logistic (4 PL) nonlinear regression model using GraphPad Prism 8 software to calculate the IC_50_. The eight more active compounds were selected to determine the mechanism of inhibition and the *K*_*i*_ values, which were calculated from four compound concentrations and five substrate concentrations (100–6.25 μM). Active enzyme concentration was determined by titration with a chloromethyl ketone irreversible inhibitor of POPTc80.^13^ All kinetic parameters were determined by nonlinear regression employing the GraphPad Prism enzyme kinetics module and Hanes-Woolf plots.^64^

#### 2.2.3 Host Cell Infection Inhibition Assay

The assay was performed as previously described by Grellier et al.,^15^ with slight modifications. Briefly, L6 cells were seeded in flasks containing RPMI medium supplemented with 5% fetal bovine serum and 1% penicillin/streptomycin (Gibco 15140122) and incubated at 37 °C in a 5% CO_2_ atmosphere. Trypomastigotes of *T. cruzi* strain Y were preincubated for 1 h at 37 °C in a culture medium containing different concentrations of inhibitors (0–200 μM, five points). Subsequently, they were added to L6 cell cultures that had been previously grown for 24h in 96-well plates (1000 cells/well) at a trypomastigote-to-host cell ratio of 30:1. After 3 h incubation at 37 °C, the wells were washed three times with inhibitor-free culture medium, and the cells were maintained for 72 h without inhibitors before fixation and staining with panoptic stain. The percentage of infected cells was determined by counting at least 200 host cells under 400-fold magnification. The inhibition of host cell invasion was calculated by comparing the percentage of infected cells in treated wells to that in untreated control wells. The concentration required to inhibit 50% of invasion (EC_50_) was determined using a nonlinear regression model applied to dose–response curves in GraphPad Prism v.8.

#### 2.2.4 Cytotoxicity Assay

L6 cells were cultured in 96-well plates at a density of 1 × 10^4^ cells/well in 100 μL of RPMI 1640 medium supplemented with 10% heat-inactivated fetal bovine serum (FBS) and 25 mM HEPES pH 7.4, maintained at 37 °C, under a 5% CO_2_ atmosphere. After 24h, the compounds were diluted, starting at a concentration of 200 μM, in 100 μL of complete culture medium per well. Subsequently, 100 μL of the diluted compounds were added to the seeded cells. After 72 h of incubation, Resazurin solution (Sigma) was added to a final concentration of 20 μM and incubated for another 4h at 37 °C. Fluorescence was then measured at 570 nm (excitation) and 595 nm (emission) using a SpectraMax M5 microplate reader (Molecular Devices, Sunnyvale, CA, USA). The cytotoxic concentrations causing 50% inhibition of cell growth (CC_50_) were determined from data obtained in two independent experiments and calculated similarly to the EC_50_, as described above.

## 3 Results and Discussion

### 3.1 POPTc80 Structure Prediction

Since the 3D structure of POPTc80 has not been experimentally resolved, it was constructed in a closed state using three approaches: (i) a homology modeling approach implemented in the SWISS-MODEL server,^29^ (ii) a threading approach implemented in the I-TASSER server,^30^ and (iii) *ab initio* method available on AlphaFold server.^31^ The 3D model from SWISS-MODEL was developed using the *Sus scrofa* prolyl oligopeptidase (PDB ID: 4AMY, resolution: 2.0 Å, sequence identity: 42.3%^65^), while the other models were generated using template-independent methods. After model building, the loops and terminal regions of the POPTc80 structures were refined using global and local geometric operators available on the GalaxyRefine server.^33,34^

**Figure 1:**
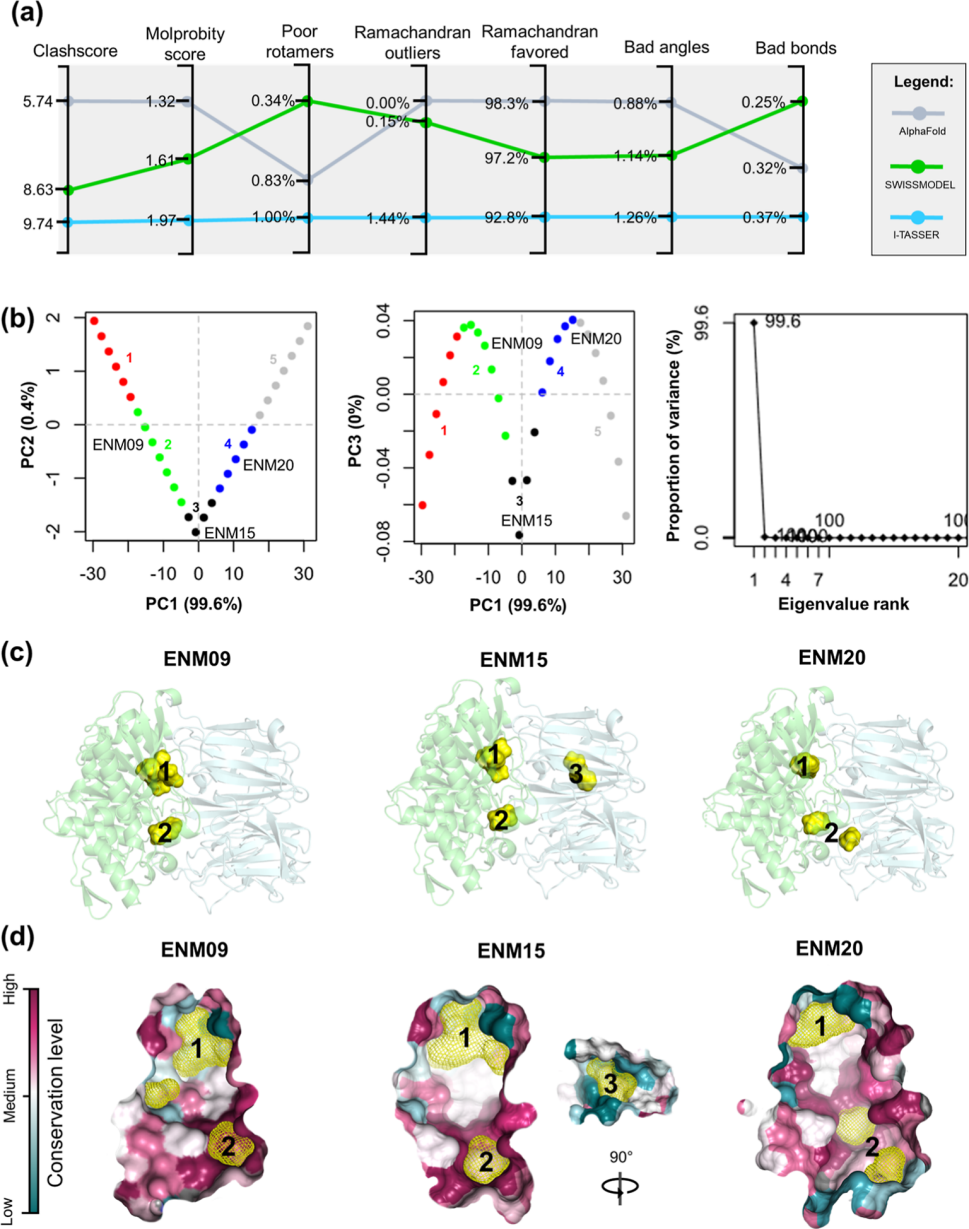
Geometrical analysis and structural insights obtained from POPTc80 conformations. (a) Statistical quality characteristics of POPTc80 models developed using different methods; (b) PCA plots illustrating conformational clusters of POPTc80 (ENM09, ENM15, and ENM20) based on collective displacements in the binding site regions. (c) 3D structural representations of the selected POPTc80 conformations, highlighting the binding pockets (1 and 2 at the domain interface and 3 in the β-propeller central tunnel) predicting using hots-spots analysis. (d) Surface representation of the predicted binding pockets with color-coded evolutionary conservation scores, indicating highly conserved regions (magenta) and less conserved regions (turquoise and white).

As shown in Figure 1a, most amino acids in the constructed POPTc80 models fall within the favored Ramachandran regions (92.8–98.3%), indicating the satisfactory quality of the torsional angles ψ and φ of the N–Cα and Cα-C bonds. Notably, the POPTc80 model developed using AlphaFold exhibited the highest geometric quality, with a Clashscore of 5.74, a MolProbity score of 1.32, 98.3% of residues in the allowed regions, and 0.0% in the disallowed regions.

The POPTc80 model (Supporting Information Figure S1a) presents an α/β-hydrolase catalytic domain containing the catalytic triad (Ser548–Asp631–His667) and a β-propeller noncatalytic domain. The α/β hydrolase domain (Figure S1b) is formed by eight β-sheet strands surrounded by eight other external α-helices. The β-propeller domain (Figure S1c) contains a set of antiparallel β-strands twisted and radially positioned around a central funnel opening. The β-propeller covers the catalytic site in a central cavity at the domain interface. The catalytic triad (Figure S1d) is composed of a nucleophile (Ser548), a proton carrier (His667), and an acid (Asp631). Mechanistically, the His667 acts as a general base to polarize and abstract the Ser548 proton, while the negatively charged Asp631 maintains the imidazole ring in a suitable position for capturing the serine proton during catalysis.^13^

### 3.2 Structural Analysis of POPTc80

Incorporating backbone flexibility into protein–ligand docking complexes is still a challenging problem.^66^ Therefore, an ENM analysis was employed to generate multiple conformations of POPTc80, incorporating backbone flexibility into the protein–ligand docking process. Subsequently, these ENM-generated conformations were analyzed to capture large-scale collective motions of the POPTc80 model, which may reflect uncertainties in the 3D coordinates and local flexibility associated with ligand binding. Figure 1b shows the low-dimensional “conformational plot”, displaying POPTc80 conformations projected onto the first principal components: PC1 vs PC2 and PC1 vs PC3 of the PCA analysis. The first and second PCs explained 100% of the total variability of conformational data.

As shown in Figure 1c, three representative POPTc80 conformations (ENM09, ENM15, and ENM20) were prioritized due to significant variations detected in the hot-spot analysis. These conformations displayed notable changes in the shape and volume of the binding pockets, revealing two consensus pockets located at the domain interface (pockets 1 and 2) and one pocket within the central tunnel of the β-propeller (pocket 3). Notably, in the ENM09 conformation, pocket 1 presented a larger and more pronounced hotspot, suggesting an enhanced potential for ligand interactions in this region. Conversely, the ENM20 conformation revealed a more prominent hotspot in pocket 2, indicating that this site may play a more critical role in binding under certain conformational states. These observations underscore the dynamic nature of POPTc80s binding pockets and highlight the importance of conformational variability for prospective structure-based investigations.

A previous study suggests that POPTc80′ substrates could access the active site via the β-propeller central tunnel (Figure S1c).^67^ However, the β-propeller domain of POPs was found to be too rigid for modulating catalysis.^68^ Therefore, pocket 3 was not considered relevant for modulating POPTc80 inhibitory activity. On the other hand, substrates can access the active site through an open conformation (Figure S1e) involving a flexible region comprising β-propeller loop A (residues 188–209) and α/β hydrolase loops B (residues 571–599) and C (residues 663–672) at the domain interface. The X-ray structure of *Pyrococcus furiosus* prolyl oligopeptidase (PDB ID: 5T88, resolution: 1.90 Å) in an open state supports this hypothesis, suggesting that this conformation is readily accessible to substrates.^69^ In contrast, the closed conformation (Figure S1f) is the preferred state in POP X-ray structures cocrystallized with competitive inhibitors.^70,71^

Subsequently, a ConSurf analysis was employed to estimate the degree of conservation of POPTc80s amino acids and predict the biological significance of the predicted binding sites, as functionally important regions tend to be more evolutionarily conserved than other positions. Figure 1d breaks down the interface domain (pocket 1: Met545, Ile683, Tyr684, Ser841, Ser480, Gln470, and Ile676) and β-propeller (pocket 3: Ser76, Asn77, Ser79. Tyr86, Leu99, Tyr130, Ala131, Trp132, Leu139, and Phe381) pockets, which includes poorly or moderately conserved residues. The lower conservation level of pocket 3 presumably reflects differences in the evolutionary pressures, suggesting that pocket 3 is not biologically relevant. In contrast, the highly conserved nature of pocket 2 of the interface domain (Cys255, Phe173, Phe470, Ser548, Asn549, Val574, Ile585, Ala588, Trp589, Tyr593, Val634, and Arg633) suggests that a unique combination of amino acids is required to carry out the enzymatic activity. The high conservation state of residues in pocket 2 results from the negative selection against mutations in these positions, as such mutations may result in loss of function.^72^ Therefore, we consider pocket 2 the most attractive binding site for prospective molecular docking studies.

### 3.3 Validation of Docking Protocol

**Figure 2:**
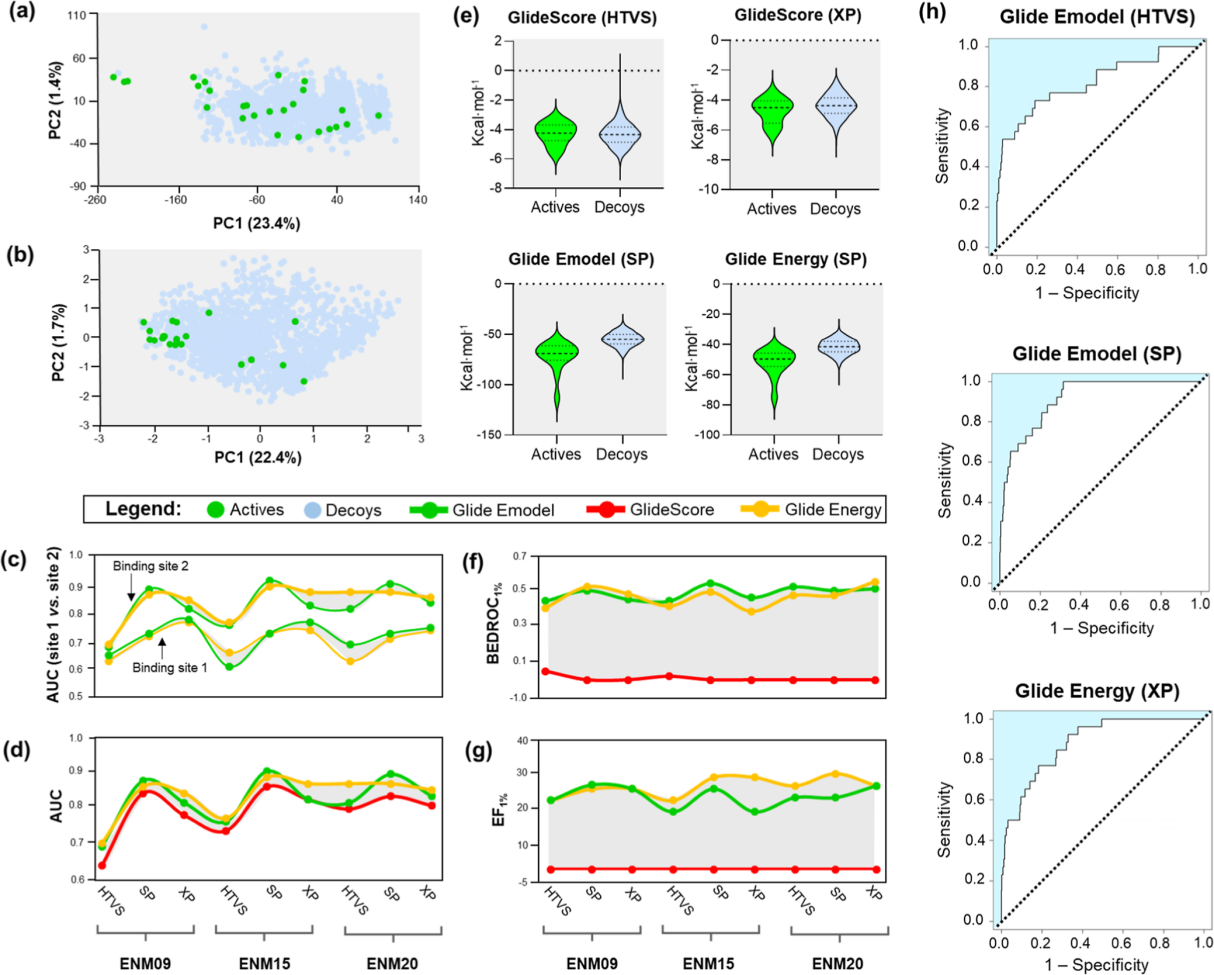
PCA plot of the first two principal components based on (a) physicochemical descriptors and (b) ECFP4 features of actives and decoys. (c) AUC values from docking of actives and decoys onto pockets 1 and 2. (d) AUC values from GlideScore, Glide Energy, and Glide Emodel scores of docking (HTVS, SP, and XP modes) onto pocket 2. (e) Violin plots of the distributions of GlideScore, Glide Energy and Glide Emodel scores (kcal/mol) for actives and decoys. BEDROC (f) and EF (g) values obtained from GlideScore, Glide Energy, and Glide Emodel scores of docking (HTVS, SP, and XP) onto pocket 2. (h) ROC curves for the best HTVS, SP, and XP docking protocols.

Docking scores are widely acknowledged to exhibit low correlation with experimental properties. Given this limitation, the enrichment rates of the docking protocol were evaluated by assessing its ability to rank a higher proportion of actives near the top of the ranked list compared to random selection.^21,22^ Here, the enrichment rates of docking were accessed using a benchmarking data set composed of 24 POPTc80 inhibitors (actives) and 864 decoys. As shown in Figure 2a, actives share the same physical-chemical space of decoys onto user-defined first two PCs. However, the actives have a limited distribution in the chemical space represented by ECFP4 features, indicating structural dissimilarity to the decoys (Figure 2b). These observations suggest that benchmark data set is suitable for assessing the docking protocol.

The enrichment rates of docking were evaluated across different grids (i.e., pockets 1 and 2), POPTc80 conformations (i.e., ENM09, ENM15, and ENM20), precision modules (HTVS, SP, and XP), and scoring functions (i.e., GlideScore, Glide Emodel, and Glide Energy). Details of docking validation are provided in Tables S3–S8. As shown in Figure 2c, the AUC values of docking onto pocket 2 outperformed docking in pocket 1, indicating that this is likely the preferred site for binding competitive inhibitors. This observation corroborates the binding modes of cocrystallized ligands of homologous POPs deposited in the PDB.^70,71^ Therefore, subsequent docking analyses will focus exclusively on pocket 2, given its superior performance in enrichment rates and alignment with known binding modes. In contrast, Figure 2d demonstrates that all scoring functions produced similar AUC values. Although AUC is important for evaluating how effectively active compounds are ranked compared to randomly selected decoys, it does not adequately reflect the relative positioning of actives and decoys within the top *x*% of the ranked list. For example, the violin plots in Figure 2e suggest a near-random distribution of actives and decoys, as both exhibit similar GlideScore distributions. However, the analysis reveals that Glide Energy and Glide Emodel functions are more effective in ranking actives above decoys.

To address the ’early recognition’ issue, EF and BEDROC were utilized in the enrichment rate analyses. Figure 2f,g illustrate that both the Glide Energy and Glide Emodel functions consistently achieve higher EF and BEDROC values across all tested protocols. In contrast, the GlideScore function displays EF and BEDROC values near zero. Docking performed using the ENM20 conformation and the Glide Emodel function exhibited the best statistical performance when tested with the HTVS protocol (AUC = 0.82, EF_1%_ = 26.9, and BEDROC_1%_ = 0.53). In contrast, docking using the ENM15 conformation with the Glide Emodel function yielded the best performance for the SP protocol with AUC = 0.92, EF_1%_ = 29.6, and BEDROC_1%_ = 0.55. Additionally, docking with the ENM20 conformation and Glide Energy function showed the highest statistical parameters among those tested with the XP protocol (AUC = 0.86, EF1% = 26.9, and BEDROC1% = 0.56). Figure 2h presents the ROC curves of the best-performing docking protocols. Based on these results, the performance of the docking protocols can be ranked as follows: ENM20 > ENM15 > ENM09. For the scoring functions, the enrichment efficacy ranks as follows: Glide Emodel ≥ Glide Energy > GlideScore. These findings indicate that the docking protocol is statistically robust and suitable for further use in VS pipelines.

### 3.4 Validation of Shape-Based Models

**Figure 3:**
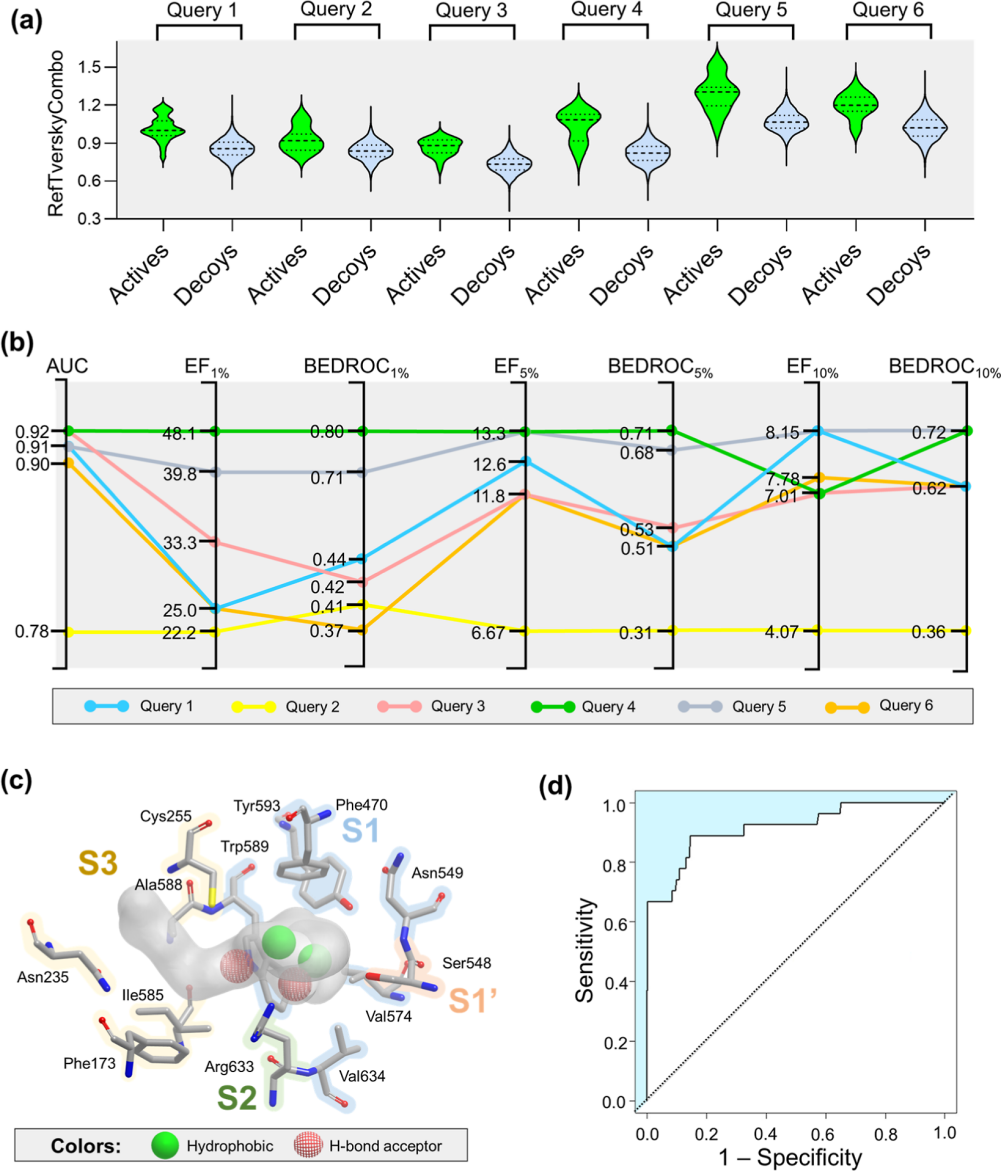
(a) Violin plots showing the distributions of RefTverskyCombo scores for actives and decoys ranked using six shape-based models. (b) Enrichment rates of developed shape-based models. (c) Schematic overview of the best shape-based model (query 4). The amino acid residues of S1, S1′, S2, and S3 regions are represented in the shape-based model coordinates to rationalize the color selection, which means key pharmacophoric features for inhibiting POPTc80. (d) ROC curve of the best shape-based model ranked by the RefTverskyCombo scoring function.

Shape-based models (Figure S2) were developed to identify novel POPTc80 inhibitors, based on the principle that compounds with similar shapes are likely to exhibit similar biological properties. Initially, the docking poses (conformations) of six known POPTc80 inhibitors were used as queries to develop the shape-based models. Actives and decoys were then aligned to each query through a rigid-body optimization process, maximizing the overlap volume between them. The performance of the shape-based models in distinguishing actives from decoys was subsequently evaluated using enrichment metrics. The results of the enrichment evaluation for the shape-based models are presented in Figure 3.

The preliminary analysis of RefTverskyCombo scores using violin graphs (Figure 3a) suggests that shape-based models rank actives better than decoys. The AUC, EF, and BEDROC were employed in the analyses to access the enrichment rates of the shape-based models. As shown in Figure 3b, all models present AUC values ranging between 0.72–0.92, having good to excellent discriminatory power for distinguishing between actives and inactives. Among them, the model developed with query 4 (see Table S1, IC_50_ = 0.025 μM^19^) showed the best statistical performance, with EF values of 48.1, 13.3, and 7.01; and BEDROC values of 0.80, 0.71, and 0.72 at the top 1%, 5% and 10% of the ranked database, respectively. These EF values reflected that the VS is highly effective, especially in the top 1% and 5%. Moreover, BEDROC for the top 1% was excellent, and for the top 5% and 10% was good. Given these results, we chose the top 1% of the ranked database to select compounds for experimental validation, ensuring the highest likelihood of success. The best shape-based model features included two hydrophobic groups (green spheres) and two hydrogen-bond acceptors (red grid spheres), which represent key pharmacophoric features for inhibiting POPTc80^60,73,74^ (Figure 3c). According to docking, the hydrogen-bond acceptor features can form interactions with Trp589 and Arg633 of S1 and S2 regions, respectively. Furthermore, the hydrophobic features can form van der Waals interactions with residues of the S1 region (Trp589, Tyr593, and Phe470). The ROC curve for this shape-based model is presented in Figure 3d, further highlighting its predictive performance.

### 3.5 Virtual Screening

**Figure 4:**
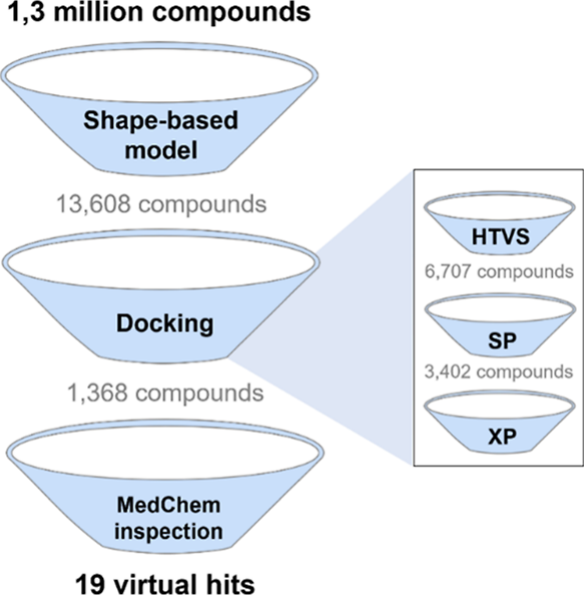
Virtual screening pipeline used for prioritizing potential POPTc80 inhibitors. The pipeline includes key steps such as shape-based filtering, molecular docking using multiple precision protocols (HTVS, SP, XP), and medicinal chemistry inspection based on binding interactions and physicochemical properties (i.e., cLogS > −5.0; cLogP >0.0 and <4.0).

The virtual screening (VS) of new POPTc80 inhibitors followed the pipeline shown in Figure 4. Initially, approximately 1.3 million compounds from the ChemBridge database were downloaded and prepared at neutral pH. The best shape-based model was then used to filter and prioritize compounds with potential POPTc80 inhibitory activity. The top 1% of compounds (13,680 structures) from the shape-based filtering were subjected to sequential docking calculations (HTVS > SP > XP) using the ENM20 conformation and Glide Emodel scoring function. Poses that formed hydrogen-bond interactions with Trp589 and Arg633 (S1 and S2 regions, respectively) and van der Waals interactions with Trp589, Tyr593, and Phe470 (S1 region) were prioritized for further analysis. Finally, a medicinal chemistry inspection was conducted to select structurally diverse hits with acceptable physicochemical profiles (i.e., cLogS > −5.0; cLogP >0.0 and <4.0). This process led to the selection of 15 putative hits for experimental validation.

A Tanimoto structural similarity (Ts) analysis using Extended-Connectivity Fingerprints with radius 4 (ECFP4) revealed that our POPTc80 inhibitors have distinct molecular scaffolds (see details in Table S9) compared to previously reported POPTc80 inhibitors (Ts < 0.66) and known trypanocidal compounds (Ts < 0.45). These compounds’ unique structural features and interaction patterns suggest they occupy a novel chemical space for POPTc80 inhibition.

### 3.6 POPTc80 Inhibition and Kinetic Assays

**Figure 5:**
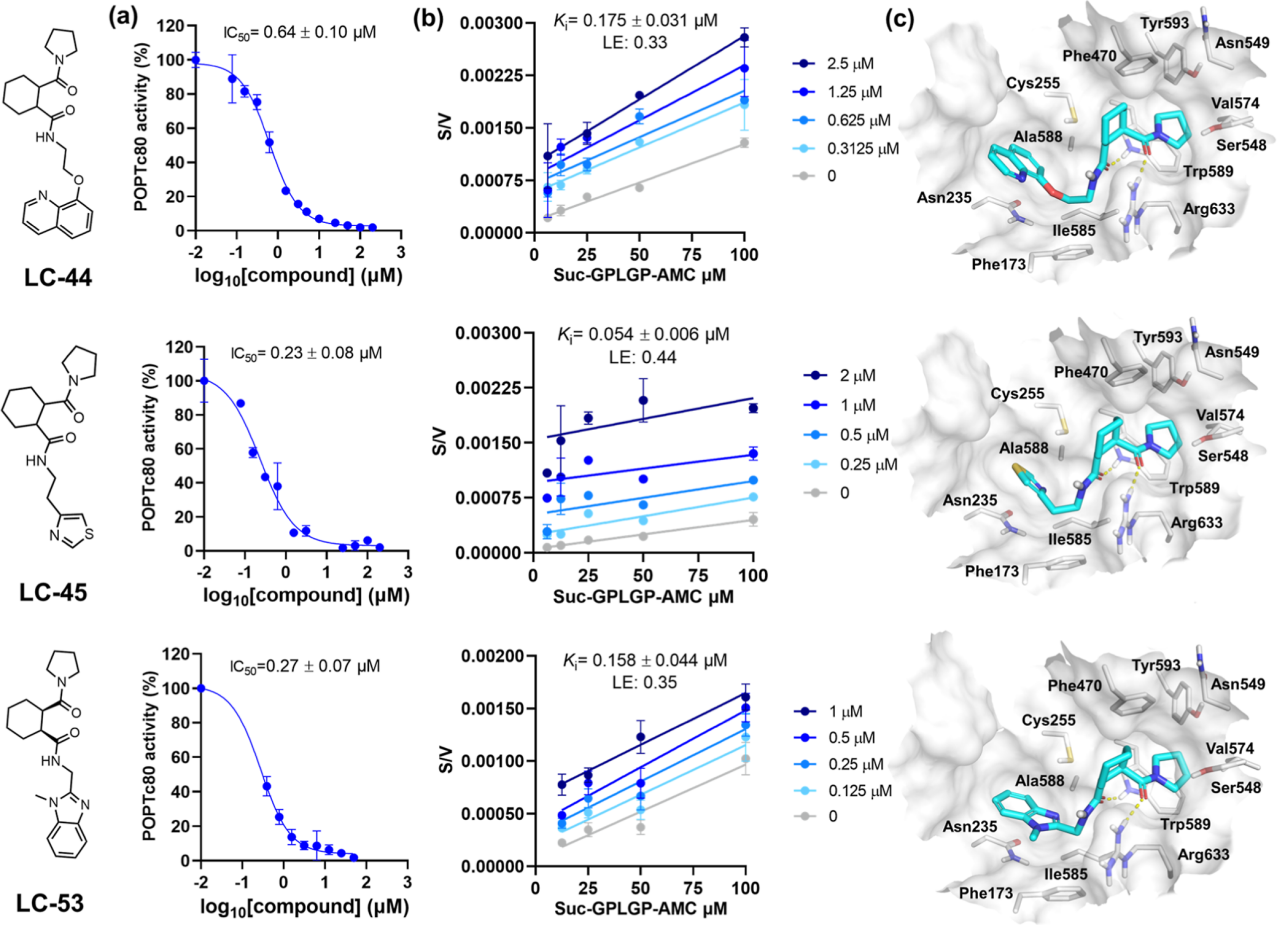
Dose–response curves (a), Hanes–Woolf plots (b), and predicted binding modes (c) of compounds LC-44, LC-45, and LC-53 on POPTc80.

The 19 putative hits were in vitro evaluated for their inhibitory activity against the POPTc80 enzyme (Table 1). Among them, 10 compounds showed inhibition, with IC_50_ values ranging from 0.23 to 1.21 μM. Notably, compounds LC-45 (0.23 ± 0.08 μM), LC-46 (0.40 ± 0.24 μM), LC-53 (0.27 ± 0.07 μM), and LC-55 (0.44 ± 0.08 μM) exhibited the best IC_50_ values. Details of dose–response curves are available in Figure 5 and Supporting Information Figure S3.

**Table 1:**
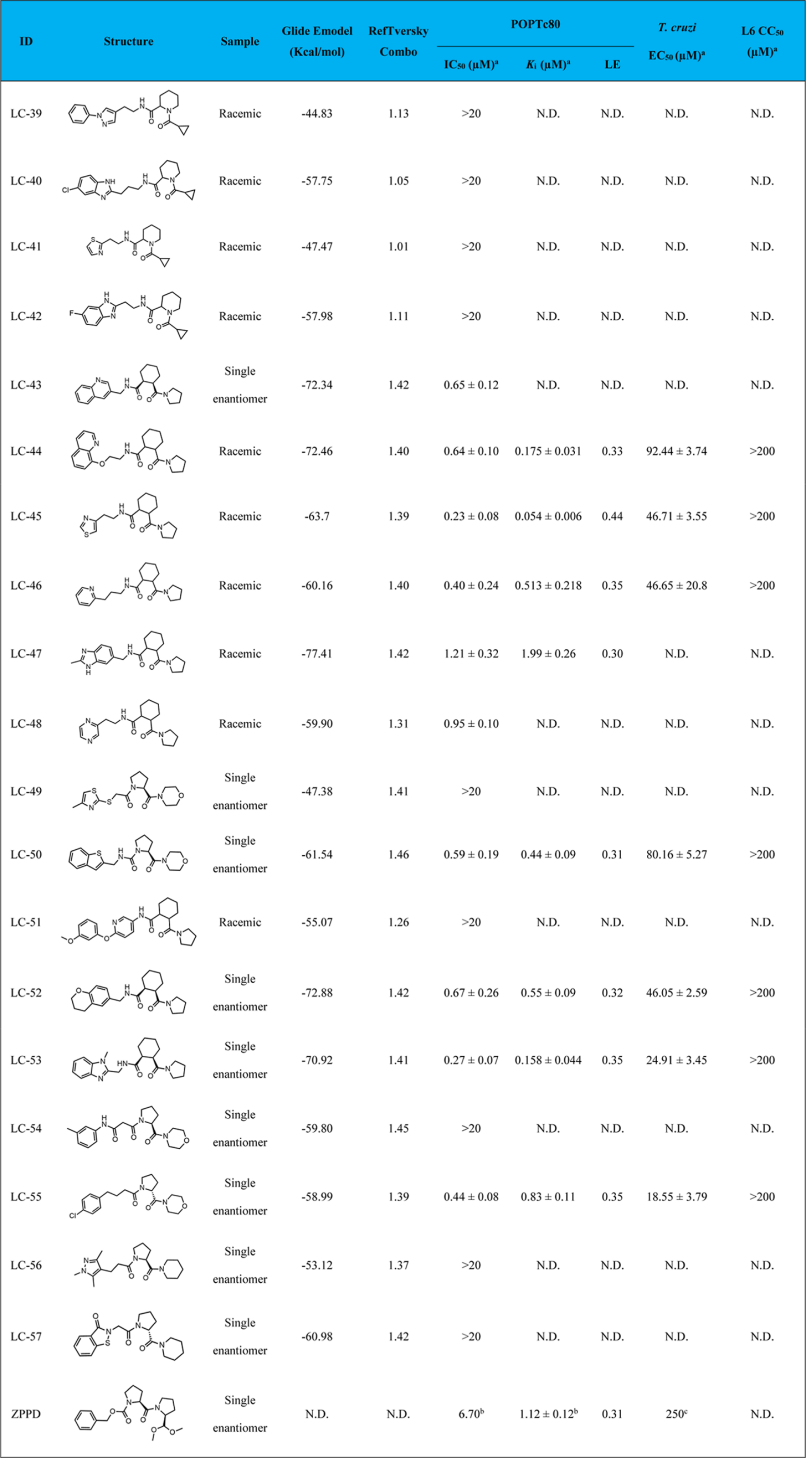
Computational Scores and In Vitro Activities of Prioritized Compounds against POPTc80^a^^,^^b^^,^^c^

a N.D., not determined; values were expressed as means ± SD of three replications; LE, ligand efficiency (kcal/mol per heavy atoms); ZPPD, Z-P-prolinal dimethylacetal.

b Inhibition data extracted from.^75^

c Phenotypic data extracted from.^15^

To further characterize the mechanism of inhibition of the most potent hits, kinetic studies were conducted using the Michaelis–Menten kinetics protocol and Hanes–Woolf plot to determine the type of inhibition. As shown in Table 1, tested compounds presented *K*_*i*_ values ranging from 0.054 to 1.99 μM. Notably, LC-44 (0.175 ± 0.031 μM), LC-45 (0.054 ± 0.006 μM), and LC-53 (0.158 ± 0.044 μM) showed the best *K*_*i*_ values. Compared to the reference inhibitor Z-P-prolinal dimethylacetal (ZPPD, *K*_*i*_ = 1.12 ± 0.12 μM),^75^ the tested compounds, especially LC-45, demonstrated significantly enhanced inhibitory affinities. Moreover, all active hits exhibited ligand efficiencies equal to or greater than the reference compound’s, with LE values ranging from 0.31 to 0.44 kcal/mol per heavy atom. This further emphasizes their potential as superior inhibitors of POPTc80.

The kinetic analysis further revealed a competitive inhibition. As illustrated by the Hanes–Woolf plots in Figure 5 and Supporting Information S3, the y-intercepts of the regression lines shift while the slopes remain unchanged, confirming a competitive inhibition mechanism. This finding suggests that the test compounds inhibit POPTc80 by directly competing with the substrate to access the enzyme’s active site. These results align with structure-based VS predictions, supporting the hypothesis that the prioritized inhibitors bind to the substrate-binding pocket, effectively impairing the enzyme’s catalytic activity.

### 3.7 Structure–Activity Relationship of POPTc80 Inhibitors

Given the promising IC_50_ and *K*_*i*_ values, docking poses of the identified inhibitors were carefully analyzed to elucidate the molecular basis for POPTc80 inhibition, providing insights into critical binding interactions and the structural features underlying their activity. As shown in Figure 5, the carbonyl groups of the inhibitors (compounds LC-44, LC-45, and LC-53) adopt a *cis*-like configuration, forming critical hydrogen bonds with Trp589 and Arg633 in the S1 and S2 regions, respectively. No electrophilic group is positioned near Ser548 of the catalytic triad, indicating a low likelihood of covalent inhibition. Furthermore, the cyclohexyl and pyrrolidin-1-yl moieties stablish van der Waals interactions with Trp589, Tyr593, and Phe470 within the S1 region. On the other hand, the aromatic and primarily heteroaromatic rings are deeply buried in the S3 pocket, where they form stabilizing van der Waals interactions with Ala588 and electrostatic interactions with Cys255 and Asn235, contributing to the overall binding affinity.

**Figure 6:**
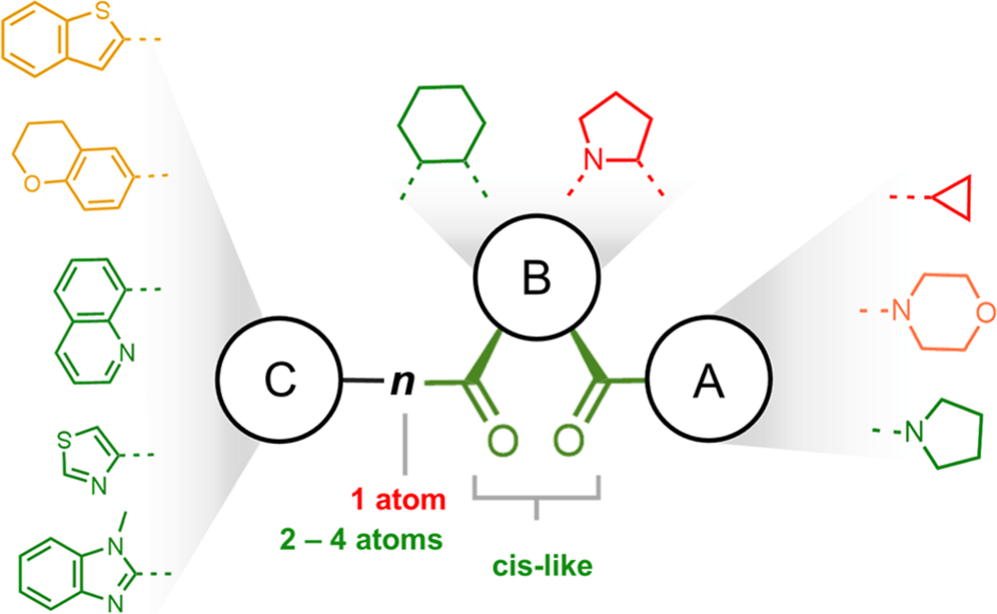
SAR rules derived from the tested compounds. Green moieties represent substituents that enhance the inhibitory activity against POPTc80, orange moieties exhibit neutral or minimal contribution to inhibition, and red moieties correspond to substituents that diminish inhibitory potency.

Based on the computational and experimental insights, preliminary structure–activity relationship (SAR) rules were developed for the tested compounds (Figure 6). The cyclopropyl substituent at position A negatively impacts inhibitory activity, while the morpholine-4-yl group does not significantly enhance potency. In contrast, the pyrrolidine-1-yl substituent at position A demonstrates the most favorable activity profile among the tested compounds. Regarding position B, six-membered rings, particularly the cyclohexyl moiety, confer the highest potency, whereas the pyrrolidine-1-yl group at this position negatively affects activity. Furthermore, the carbonyl groups connected to the cyclohexyl moiety are crucial for maintaining inhibitory potency, with both carbonyls required to adopt a *cis*-like orientation due to the chiral center. This highlights their essential role in interacting with the binding site. Conversely, compounds featuring flexible alkyl or ether linkers, typically consisting of 2 to 4 atoms, connecting rigid moieties have shown the most favorable inhibition profiles. The flexibility of these linkers enables the compounds to adopt optimal conformations within the S3 pocket, enhancing their ability to interact effectively with the binding site. Additionally, the heteroaromatic rings in these compounds are often deeply buried within the hydrophobic S3 pocket (see Figures 5 and S3). The linker allows these heteroaromatic rings to coordinate between Asn235 and Cys255, forming a stable polar interaction, further contributing to the overall binding affinity.

### 3.8 Inhibition of the Host Cell Entry by POPTc80 Inhibitors

**Figure 7:**
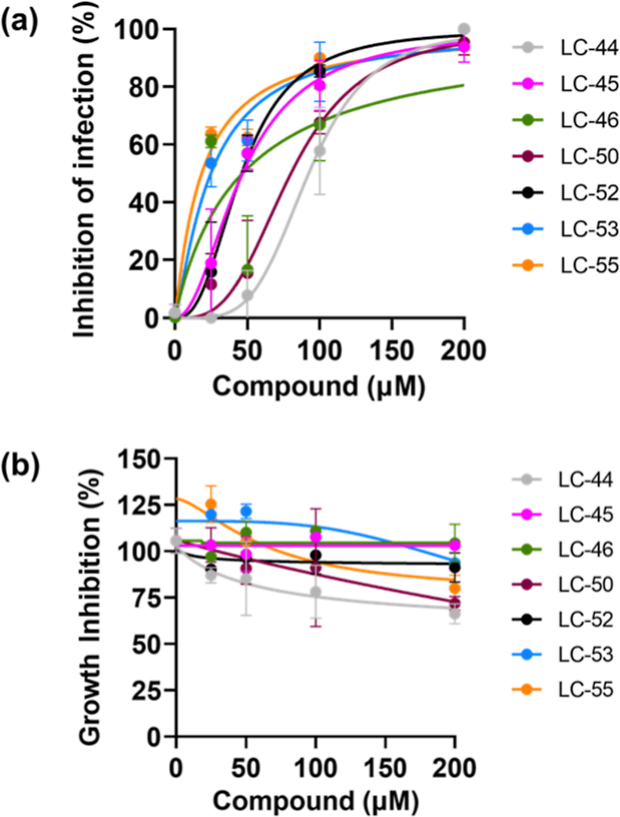
(a) Effect of pretreatment with POPTc80 inhibitors on trypomastigote infection in L6 cells. *T. cruzi* trypomastigotes were incubated with the compounds for 1 h and subsequently used to infect host cells for 3 h. The inhibition of parasite invasion was evaluated by quantifying intracellular amastigotes in stained cells. (b) Cytotoxicity against L6 cells were seeded at 1 × 10^4^ cells/well and then treated 72 h with various concentrations of the compounds.

Based on our previous observation that POPTc80 is secreted by infective trypomastigotes and that POPTc80-specific inhibitors block parasite entry into host cells,^13,15^ we assessed this process using our most potent inhibitors. Parasites were preincubated with the inhibitors for 1h and subsequently incubated with L6 cells for 3 h in the presence of the same inhibitors. Following this incubation, the L6 cells were thoroughly washed to remove any remaining parasites and inhibitors and then maintained in culture for 72 h. Figure 7a illustrates the concentration-dependent inhibition of trypomastigote entry into host cells observed for all tested inhibitors, with EC_50_ values ranging from 16.22 μM (LC-55) to 92.45 μM (LC-44) (Table 1). The three most potent inhibitors (LC-46, LC-55, and LC-53) exhibited an EC_50_ comparable to that of inhibitor 4a (15 μM), the most effective specific POPTc80 inhibitor characterized by our group.^15^ It should be noted that in this experiment, the highest EC_50_ value (LC-44, 92.45 μM) is 2.7 times lower than the EC_50_ value of the prototype inhibitor for human POP (ZPPD, 250 μM;^15^). All tested compounds exhibited low cytotoxicity (>200 μM) against L6 cells (Table 1 and Figure 7b), consistent with the profile of mammalian POP inhibitors used in vivo.^76^

### 3.9 *In Silico* ADMET Profile

An *in silico* analysis of pharmacokinetic and toxicological properties (ADMET) was performed using ADMETLab v.3.0^77^ to evaluate the identified hits as potential candidates for prospective hit-to-lead investigations. As shown in Table S10, our hits revealed favorable drug-like properties and improved safety profiles compared to benznidazole (Table S10). The selected hits displayed strong desirability based on the drug-likeness QED filter, showing no alerts for assay interference. They demonstrated high permeability in Caco-2 cells, along with excellent intestinal absorption. Most hits demonstrated adequate plasma protein binding and limited permeability across the blood–brain barrier. Metabolic profiling revealed that the hits were predominantly metabolized by CYP2D6 and CYP3A4, with a notable potential to inhibit these enzymes, consistent with their predicted low stability in human liver microsomes. Nonetheless, plasma clearance values varied from low to intermediate. Toxicological predictions revealed a low to moderate potential for hERG channel blockade and AMES mutagenicity. However, none of the hits exhibited structural alerts associated with these risks, indicating a favorable toxicological profile. Acute oral toxicity levels varied between low and intermediate categories. These findings underscore the compounds’ potential as promising candidates for hit-to-lead optimization.

## 4 Conclusions

This study employed a comprehensive structure-based virtual screening approach to successfully identify novel inhibitors of POPTc80, a key enzyme in *T. cruzi* infection. Through the integration of shape-based modeling and molecular docking, 19 compounds were prioritized, with experimental validation identifying 10 potent inhibitors exhibiting nanomolar to micromolar activity. Kinetic studies confirmed a competitive inhibition mechanism for these compounds, consistent with the structure-based pipeline. In addition, these compounds likely form fundamental interactions with Trp589, Arg633, and other residues critical for POPTc80 function. Furthermore, the most promising compounds—LC-44 (*K*_*i*_ = 0.175 μM), LC-45 (*K*_*i*_ = 0.054 μM), LC-46 (*K*_*i*_ = 0.513 μM), LC-50 (*K*_*i*_ = 0.44 μM), LC-53 (*K*_*i*_ = 0.158 μM), and LC-55 (*K*_*i*_ = 0.83 μM)—demonstrated the ability to significantly impair *T. cruzi* cell entry in a phenotypic infection model, further reinforcing the therapeutic relevance of POPTc80 as a target. All tested compounds also exhibited low cytotoxicity against mammalian cells, supporting their selectivity and favorable safety profile. Taken together, these results establish our hits as valuable chemical scaffolds for hit-to-lead optimization, offering a promising avenue for developing novel anti-*T. cruzi* agents that target a critical step in parasite invasion.

## Data Availability

Data and Software Availability: all the data are available on the GitHub repository (https://github.com/TaoLen/POPTc80).

## Supporting Information Available

Additional details and data on the in silico ADMET prediction protocol, the chemical similarity analysis protocol, the parameters and queries explored in molecular docking and shape-based models, the enrichment rates from molecular docking and shape-based models, additional similarity analysis, the in silico ADMET profiles of hit compounds, and the ^1^H NMR and LC–MS spectra for test compounds are provided (PDF)

## Abbreviations

3D: three dimensional
ANN: anisotropic network model
AUC: area under curve
BEDROC: bolzmann enhanced discrimination
EF: enrichment factor
ENM: elastic network models
EC50: half-maximal effective concentration
IC50: half-maximal inhibitory concentration
*K*_*i*_: inhibition constant
LogP: partition coefficient
PAINS: pan-assay interference compounds
PDB: protein data bank
POPTc80: *Trypanosoma cruzi* prolyl oligopeptidase 80
PCA: principal component analysis
RIE: robust initial enhancement
RMSD: root-mean-square deviation
ROC: receiver operating characteristic
*T. cruzi*: *Trypanosoma cruzi*
VS: virtual screening
