# Supplementary material for: Structure-Based Identification of Non-covalent Prolyl Oligopeptidase 80 Inhibitors Targeting Trypanosoma cruzi Cell Entry
Source: J Chem Inf Model. 2025 Feb 26;65(5):2636–49. doi: 10.1021/acs.jcim.4c02152 (PMC11898053; doi:10.1021/acs.jcim.4c02152)
Supplement: Supplementary file 1 — ci4c02152_si_001.pdf [file ci4c02152_si_001.pdf]

Supplementary Information

Structure-Based Identification of Non-Covalent Prolyl Oligopeptidase 80 Inhibitors Targeting *Trypanosoma cruzi* Cell Entry

Vinícius Alexandre Fiaia Costa[a], Flávia Nader Motta[b], Alexandra Maria dos Santos Carvalho[b], Felipe da Silva Mendonça de Melo[b], Melina Mottin[b], Sébastien Charneau[c], Philippe Grellier[d], Jaime Martins de Santana[b], Izabela Marques Dourado Bastos[b]*, Bruno Junior Neves[a]**

[a] Laboratory of Cheminformatics, Faculty of Pharmacy, Federal University of Goiás, Goiânia, Brazil

[b] Pathogen-Host Interface Laboratory, Department of Cell Biology, Institute of Biological Sciences, University of Brasilia, Brasilia, Brazil

[c] Laboratory of Biochemistry and Protein Chemistry, Department of Cell Biology, Institute of Biological Sciences, University of Brasilia, Brasilia, Brazil

[d] UMR 7245 Molécules de Communication et Adaptation des Micro-organismes, Muséum National d'Histoire Naturelle, Équipe Parasites et Protistes Libres, Paris, France

* Corresponding author: email: dourado@unb.br

** Corresponding author: email: brunoneves@ufg.br

CONTENTS

1. *In silico* ADMET prediction protocol S2
2. Chemical similarity analysis protocol S2
3. Parameters and queries explored in molecular docking and shape-based models S3
4. Enrichment rates from molecular docking and shape-based models S4
5. Additional similarity analysis and *in silico* ADMET profiles of hit compounds..................S7
6. ^{1}H NMR and LC-MS spectra for test compounds...S12
7. References S31

1. *In silico* ADMET analysis protocol

The ADMETLab v.3.0 [1] was used to predict key drug-like properties of the most potent POPTc80 inhibitors and reference drug benznidazole. The analysis included drug quality metrics (QED), potential false positives (PAINS, colloid aggregators, FLuc inhibitors), absorption parameters (Caco-2 permeability, HIA), distribution factors (PPB, BBB, Fu), metabolism indicators (CYP inhibition/substrate profiles, HLM stability), elimination markers (plasma clearance), and toxicity measures (hERG blocking, mutagenicity, and acute toxicity).

2. Chemical similarity analysis protocol

Chemical similarity analysis was performed to identify the nearest neighbors of our POPTc80 inhibitors using two reference datasets: (i) phenotypic dataset comprising over 19,000 compounds with anti-*T. cruzi* activity reported in ChEMBL database (ID: CHEMBL368) and (ii) previously reported POPTc80 inhibitors from our benchmark dataset. The analysis utilized ECFP4 fingerprints (2048 bits) derived from SMILES structures, with Tanimoto coefficients calculated to quantify the structural relationships between our active compounds and the reference molecules.

3. Parameters and queries explored in molecular docking and shape-based models

Table S1. Grid box coordinates used in Glide for molecular docking studies against POPTc80.

**POPTc80's conformations**	**Center grid box (*x*, *y* and *z* coordinates)**	
	**Binding site 1**	**Binding site 2**
**ENM09**	21.8 Å; 22.4 Å; 21.6 Å	27.7 Å; 46.7 Å; 43.0 Å
**ENM15**	23.3 Å; 23.3 Å; 23.0 Å	27.7 Å; 46.7 Å; 43.4 Å
**ENM20**	22.5 Å; 21.9 Å; 21.6 Å	27.9Å; 46.8 Å; 43.1 Å

Table S2. Chemical structures of known POPTc80 inhibitors used as queries for developing shape-based models.

**Query**	**Chemical structure**	**POPTc80 bioactivity**	**Source**
**Query 1**	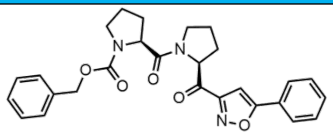	$K_i = 0.00021\ \mu M$	[2]
**Query 2**	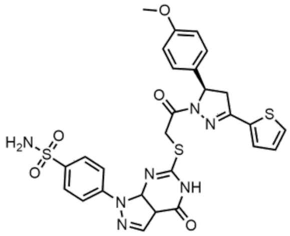	$K_i = 1.009\ \mu M$	[3]
**Query 3**	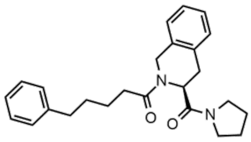	$IC_{50} = 0.014\ \mu M$	[4]
**Query 4**	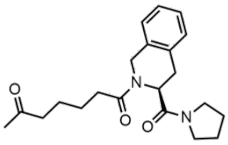	$IC_{50} = 0.025\ \mu M$	[4]
**Query 5**	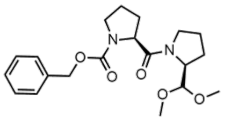	$K_i = 1.120\ \mu M$	[5]
**Query 6**	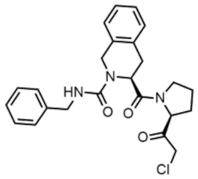	$IC_{50} = 0.0038\ \mu M$	[6]

4. Enrichment rates from molecular docking and shape-based models

Table S3. Statistical characteristics of docking at pocket 1 of the POPTc80 using the Glide Emodel scoring function.

Conformation	AUC	Top 1%		Top 5%		Top 10%	
		EF	BEDROC	EF	BEDROC	EF	BEDROC
			HTVS				
**ENM09**	0.63	14.9	0.33	5.93	0.29	4.44	0.33
**ENM15**	0.66	22.2	0.42	5.93	0.33	3.70	0.34
**ENM20**	0.63	23.1	0.39	6.92	0.33	3.85	0.35
			SP				
**ENM09**	0.72	25.9	0.40	8.15	0.39	4.56	0.42
**ENM15**	0.73	18.5	0.29	8.15	0.36	4.81	0.41
**ENM20**	0.71	26.9	0.43	9.23	0.39	4.62	0.44
			XP				
**ENM09**	0.77	22.2	0.34	8.89	0.39	5.56	0.45
**ENM15**	0.74	25.9	0.39	8.15	0.38	4.81	0.43
**ENM20**	0.74	19.2	0.37	7.69	0.38	5.00	0.43

Table S4. Statistical characteristics of docking at pocket 2 of the POPTc80 using the Glide Emodel scoring function.

Conformation	AUC	Top 1%		Top 5%		Top 10%	
		EF	BEDROC	EF	BEDROC	EF	BEDROC
			HTVS				
**ENM09**	0.68	22.2	0.45	5.19	0.32	3.70	0.34
**ENM15**	0.76	22.2	0.45	5.93	0.31	4.44	0.36
**ENM20**	0.82	26.9	0.53	10.8	0.48	5.77	0.52
			SP				
**ENM09**	0.89	25.9	0.51	10.4	0.47	5.56	0.52
**ENM15**	0.92	29.6	0.55	11.1	0.49	6.67	0.56
**ENM20**	0.91	30.8	0.51	11.5	0.50	6.54	0.57
			XP				
**ENM09**	0.82	25.9	0.46	8.15	0.41	5.19	0.47
**ENM15**	0.83	29.6	0.47	7.41	0.41	5.41	0.47
**ENM20**	0.84	26.9	0.52	9.23	0.44	5.38	0.49

Table S5. Statistical characteristics of docking at pocket 1 of the POPTc80 using the Glide Energy scoring function.

Conformation	AUC	Top 1%		Top 5%		Top 10%	
		EF	BEDROC	EF	BEDROC	EF	BEDROC
			**HTVS**				
**ENM09**	0.65	18.5	0.36	7.41	0.34	4.44	0.39
**ENM15**	0.61	22.2	0.42	7.41	0.35	4.07	0.37
**ENM20**	0.69	23.1	0.38	6.15	0.33	4.23	0.37
			**SP**				
**ENM09**	0.73	29.6	0.45	8.89	0.42	4.81	0.45
**ENM15**	0.73	25.9	0.36	8.15	0.40	5.19	0.45
**ENM20**	0.73	30.8	0.45	9.23	0.44	5.38	0.48
			**XP**				
**ENM09**	0.78	18.5	0.35	8.89	0.39	5.56	0.46
**ENM15**	0.77	22.2	0.40	10.4	0.43	5.93	0.49
**ENM20**	0.75	28.2	0.45	10.0	0.47	5.77	0.51

Table S6. Statistical characteristics of docking at pocket 2 of the POPTc80 using the Glide Energy scoring function.

Conformation	AUC	Top 1%		Top 5%		Top 10%	
		EF	BEDROC	EF	BEDROC	EF	BEDROC
			**HTVS**				
**ENM09**	0.69	22.2	0.41	7.41	0.32	4.44	0.36
**ENM15**	0.77	18.5	0.42	8.89	0.36	5.19	0.42
**ENM20**	0.81	23.1	0.53	10.0	0.47	5.77	0.52
			**SP**				
**ENM09**	0.87	27.2	0.53	9.63	0.47	5.56	0.51
**ENM15**	0.90	25.9	0.50	8.15	0.42	6.15	0.49
**ENM20**	0.88	23.1	0.48	10.0	0.45	5.38	0.50
			**XP**				
**ENM09**	0.85	25.9	0.49	8.89	0.44	5.56	0.49
**ENM15**	0.88	18.5	0.39	7.41	0.35	5.19	0.43
**ENM20**	0.86	26.9	0.56	8.46	0.46	5.00	0.49

Table S7. Statistical characteristics of docking at pocket 1 of the POPTc80 using the GlideScore function.

Conformation	AUC	Top 1%		Top 5%		Top 10%	
		EF	BEDROC	EF	BEDROC	EF	BEDROC
			HTVS				
**ENM09**	0.49	3.70	0.05	2.72	0.09	1.85	0.13
**ENM15**	0.55	0.00	0.02	2.96	0.10	1.85	0.15
**ENM20**	0.54	0.00	0.00	0.00	0.01	0.38	0.04
			SP				
**ENM09**	0.65	0.00	0.00	0.00	0.00	0.00	0.00
**ENM15**	0.64	0.00	0.00	0.00	0.00	0.00	0.01
**ENM20**	0.61	0.00	0.00	0.00	0.00	0.38	0.02
			XP				
**ENM09**	0.65	0.00	0.00	0.00	0.00	0.37	0.02
**ENM15**	0.72	0.00	0.00	0.00	0.01	0.37	0.02
**ENM20**	0.63	0.00	0.00	0.00	0.00	0.00	0.01

Table S8. Statistical characteristics of docking at pocket 2 of the POPTc80 using the GlideScore function.

Conformation	AUC	Top 1%		Top 5%		Top 10%	
		EF	BEDROC	EF	BEDROC	EF	BEDROC
			HTVS				
**ENM09**	0.62	0.00	0.00	0.00	0.01	0.37	0.03
**ENM15**	0.73	0.00	0.00	0.00	0.00	0.37	0.01
**ENM20**	0.80	0.00	0.00	0.00	0.00	0.00	0.00
			SP				
**ENM09**	0.85	0.00	0.00	0.00	0.00	0.00	0.00
**ENM15**	0.87	0.00	0.00	0.00	0.00	0.00	0.00
**ENM20**	0.84	0.00	0.00	0.00	0.00	0.00	0.00
			XP				
**ENM09**	0.78	0.00	0.00	0.00	0.00	0.00	0.00
**ENM15**	0.83	0.00	0.00	0.00	0.00	0.00	0.00
**ENM20**	0.81	0.00	0.00	0.00	0.00	0.00	0.01

5. Additional similarity analysis and *in silico* ADMET profiles of hit compounds

Table S9. Nearest neighbors of our hit compounds identified through Tanimoto similarity analysis with previously reported trypanocidal compounds and POPTc80 inhibitors.

ID	Nearest neighbor	
	T. cruzi dataset	POPTc80 dataset
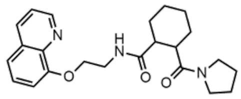 LC-44	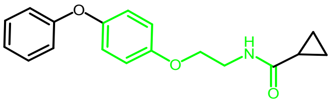 Ts = 0.32<br>IC_{50}: > 70 µM [7]	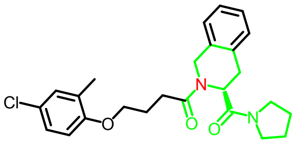 Ts = 0.29<br>IC_{50}: 0.017 µM [8]
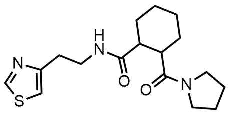 LC-45	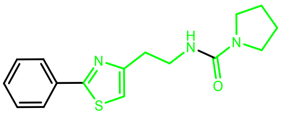 Ts = 0.37<br>IC_{50}: 3.2 µM [9]	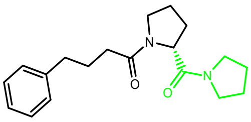 Ts = 0.27<br>IC_{50}: 12 µM [4]
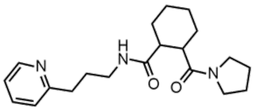 LC-46	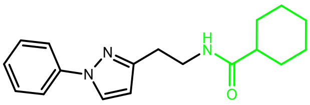 Ts = 0.37<br>IC_{50}: 0.73 µM [10]	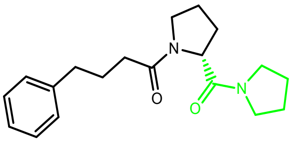 Ts = 0.34<br>IC_{50}: 12 µM [4]
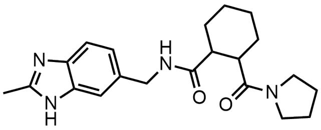 LC-47	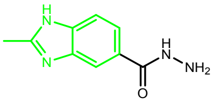 Tanimoto Similarity: 0.3333<br>IC_{50}: 393 µM [7]	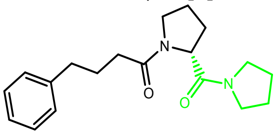 Tanimoto Similarity: 0.2639<br>IC_{50}: 12 µM [4]
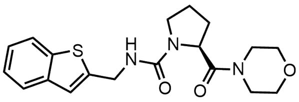 LC-50	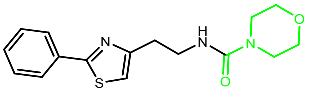 Ts = 0.33<br>IC_{50}: 3.2 µM [9]	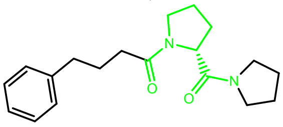 Ts = 0.37<br>IC_{50}: 12 µM [4]
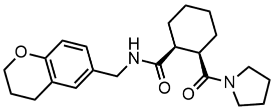 LC-52	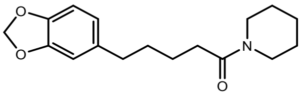 Ts = 0.31<br>IC_{50}: 19.41 µM [9]	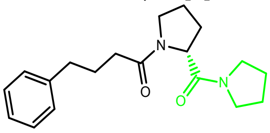 Ts = 0.27<br>IC_{50}: 12 µM [4]
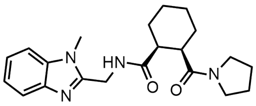 LC-53	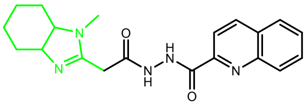 Ts = 0.42<br>IC_{50}: 5.01 µM [10]	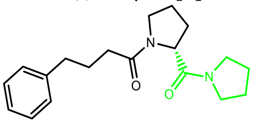 Ts = 0.29<br>IC_{50}: 12 µM [4]
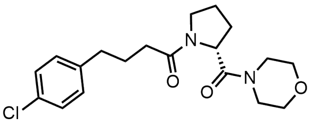 LC-55	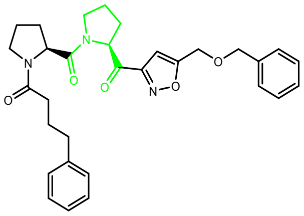 Ts = 0.44<br>ED_{50}: 4.7 µg/mL [2]	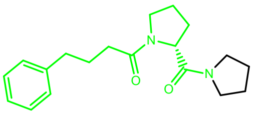 Ts = 0.65<br>IC_{50}: 12 µM [4]

Table S10. *In silico* ADMET profile of discovered POPTc80 inhibitors and reference drug benznidazole.

Property	Comment	Decision (probability/value)								
		LC-44	LC-45	LC-46	LC-47	LC-50	LC-52	LC-53	LC-55	benznidazole
QED	▪ A measure of drug-likeness based on the concept of desirability; Attractive: > 0.67; unattractive: 0.49~0.67; too complex: < 0.34.	(0.763)	(0.897)	(0.808)	(0.871)	(0.899)	(0.886)	(0.902)	(0.806)	(0.644)
PAINS	▪ Frequent hitters, Alpha-screen artifacts and reactive compound 480 substructures.	(0 alerts)	(0 alerts)	(0 alerts)	(0 alerts)	(0 alerts)	(0 alerts)	(0 alerts)	(0 alerts)	(0 alerts)
Colloidal aggregators	▪ Category 0: non-colloidal aggregators; Category 1: colloidal aggregators. The output value is the probability of being colloidal aggregators within the range of 0 to 1.	(0.066)	(0.052)	(0.029)	(0.067)	(0.118)	(0.033)	(0.230)	(0.010)	(0.187)
FLuc inhibitors	▪ Category 0: non-fLuc inhibitors; Category 1: fLuc inhibitors. The output value is the probability of being fLuc inhibitors within the range of 0 to 1.	(0.022)	(0.022)	(0.025)	(0.04)	(0.075)	(0.260)	(0.014)	(0.002)	(0.008)
Green Fluorescence	▪ Category 0: non-green fluorescence; Category 1: green fluorescence. The output value is the probability of being green fluorescence, within the range of 0 to 1.	(0.658)	(0.122)	(0.175)	(0.506)	(0.298)	(0.452)	(0.311)	(0.184)	(0.708)
Reactive compounds	▪ Category 0: non-reactive compound; Category 1: reactive compound. The output value is the probability of being reactive compound, within the range of 0 to 1.	(0.022)	(0.001)	(0.001)	(0.0)	(0.002)	(0.001)	(0.0)	(0.005)	(0.005)
Caco-2 Permeability	▪ Optimal: higher than -5.15 Log unit	(-4.677)	(-4.866)	(-4.721)	(-4.961)	(-5.232)	(-4.636)	(-4.767)	(-4.7)	(-4.698)
HIA	▪ Human Intestinal Absorption. Category 1: HIA+(HIA < 30%); Category 0: HIA-(HIA >= 30%). The output value is the probability of being HIA+	(0.011)	(0.064)	(0.021)	(0.001)	(0.147)	(0.116)	(0.071)	(0.003)	(0.0)
PPB	▪ Plasma Protein Binding Optimal: < 90%. Drugs with high protein-bound may have a low therapeutic index.	(85.623)	(30.604)	(43.813)	(51.925)	(93.127)	(64.296)	(81.153)	(92.385)	(50.416)
BBB	▪ Blood-Brain Barrier Penetration. Category 1: BBB+; Category 0: BBB-; The output value is the probability of being BBB+	(0.0)	(0.002)	(0.002)	(0.001)	(0.016)	(0.426)	(0.428)	(0.373)	(0.688)
Fu	▪ The fraction unbound in plasms. Low: <5%; Middle: 5~20%; High: > 20%	(14.851)	(63.903)	(53.594)	(44.002)	(5.598)	(32.364)	(20.702)	(6.917)	(43.056)
CYP2C9 inhibitor	▪ Category 1: Inhibitor; Category 0: Non-inhibitor. The output value is the probability of being inhibitor.	(0.212)	(0.0)	(0.0)	(0.0)	(0.0)	(0.008)	(0.0)	(0.0)	(0.001)

CYPC9 substrate	▪ Category 1: Substrate; Category 0: Non-substrate; The output value is the probability of being substrate.	(0.576)	(0.006)	(0.0)	(0.001)	(0.976)	(0.784)	(0.0)	(0.018)	(0.0)
CYP2D6 inhibitor	▪ Category 1: Inhibitor; Category 0: Non-inhibitor; The output value is the probability of being inhibitor.	(0.0)	(0.0)	(0.0)	(0.002)	(0.002)	(0.001)	(0.002)	(0.096)	(0.0)
CYP2D6 substrate	▪ Category 1: Substrate; Category 0: Non-substrate; The output value is the probability of being substrate.	(0.999)	(0.962)	(0.856)	(0.011)	(0.966)	(1.0)	(0.274)	(0.0)	(0.0)
CYP3A4 inhibitor	▪ Category 1: Inhibitor; Category 0: Non-inhibitor; The output value is the probability of being inhibitor.	(0.756)	(0.75)	(0.013)	(0.0)	(0.0)	(0.012)	(0.0)	(0.544)	(0.001)
CYP3A4 substrate	▪ Category 1: Substrate; Category 0: Non-substrate; The output value is the probability of being substrate.	(0.703)	(0.422)	(0.991)	(0.998)	(0.992)	(0.989)	(1.0)	(0.967)	(0.0)
HLM Stability	▪ Human liver microsomal (HLM) stability. Category 0: stable+ (HLM > 30 min); Category 1: unstable- (HLM ≥ 30 min). The output value is the probability of human liver microsomal instability, where a value closer to 1 indicates a higher likelihood of instability. The range is between 0 and 1.	(0.841)	(0.998)	(0.678)	(0.045)	(0.076)	(0.999)	(0.427)	(0.018)	(0.001)
CL_{plasma}	▪ The unit of predicted CLplasma penetration is ml/min/kg. >15 ml/min/kg: high clearance; 5-15 ml/min/kg: moderate clearance; < 5 ml/min/kg: low clearance.	(5.626)	(5.617)	(5.786)	(4.611)	(5.239)	(4.77)	(3.91)	(6.305)	(5.696)
hERG Blockers	▪ Molecules with IC_{50} ≤10 µM or ≥ 50% inhibition at 10 µM were classified as hERG+ (Category 1); while molecules with IC_{50} >10 µM or < 50% inhibition at 10 µM were classified as hERG - (Category 0). The output value is the probability of being hERG+, within the range of 0 to 1.	(0.275)	(0.168)	(0.241)	(0.497)	(0.405)	(0.537)	(0.595)	(0.631)	(0.312)
AMES Mutagenicity	▪ AMES Toxicity. Category 1: Ames positive (+); Category 0: Ames negative (-). The output value is the probability of being toxic.	(0.67)	(0.538)	(0.548)	(0.773)	(0.605)	(0.553)	(0.528)	(0.25)	(0.91)
Rat Oral Acute Toxicity	▪ Rat Oral Acute Toxicity. Category 0: low-toxicity, > 500 mg/kg; Category 1: high-toxicity; < 500 mg/kg; The output value is the probability of being toxic, within the range of 0 to 1.	(0.087)	(0.079)	(0.045)	(0.302)	(0.353)	(0.268)	(0.395)	(0.25)	(0.211)

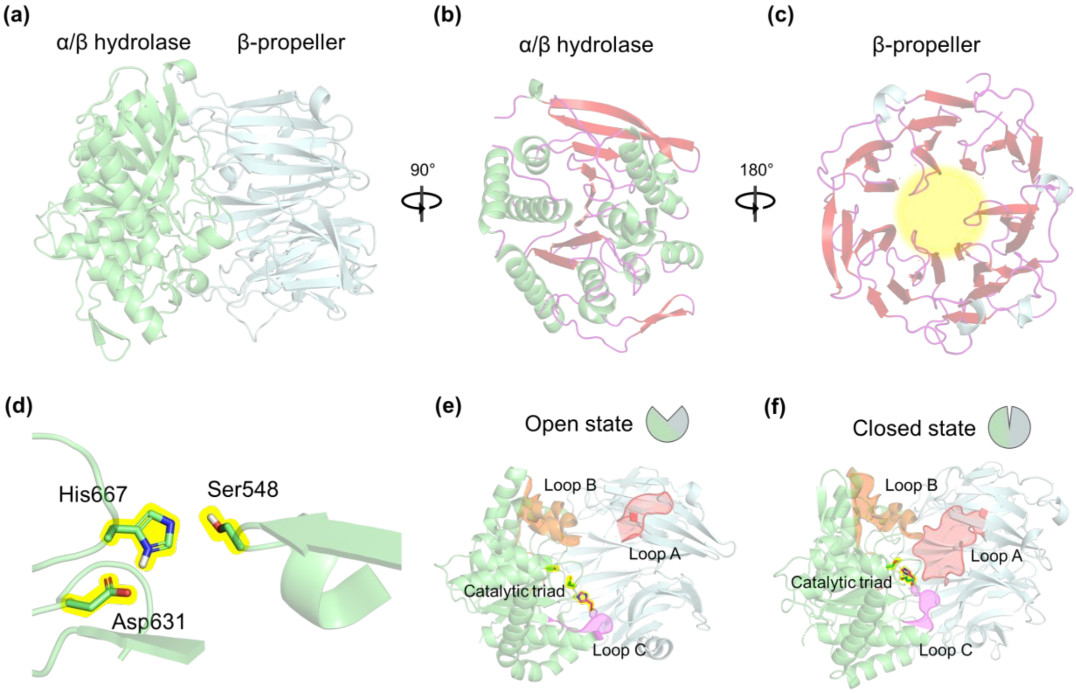

Figure S1. The 3D structures of POPs in the different conformational states: (e) the opened [obtained from X-ray structure of *Pyrococcus furiosus* prolyl oligopeptidase (PDB ID: 5T88) [11], and (f) the closed [POPTc80 model] loops A, B, and C occupying the active site in domain interface of protease.

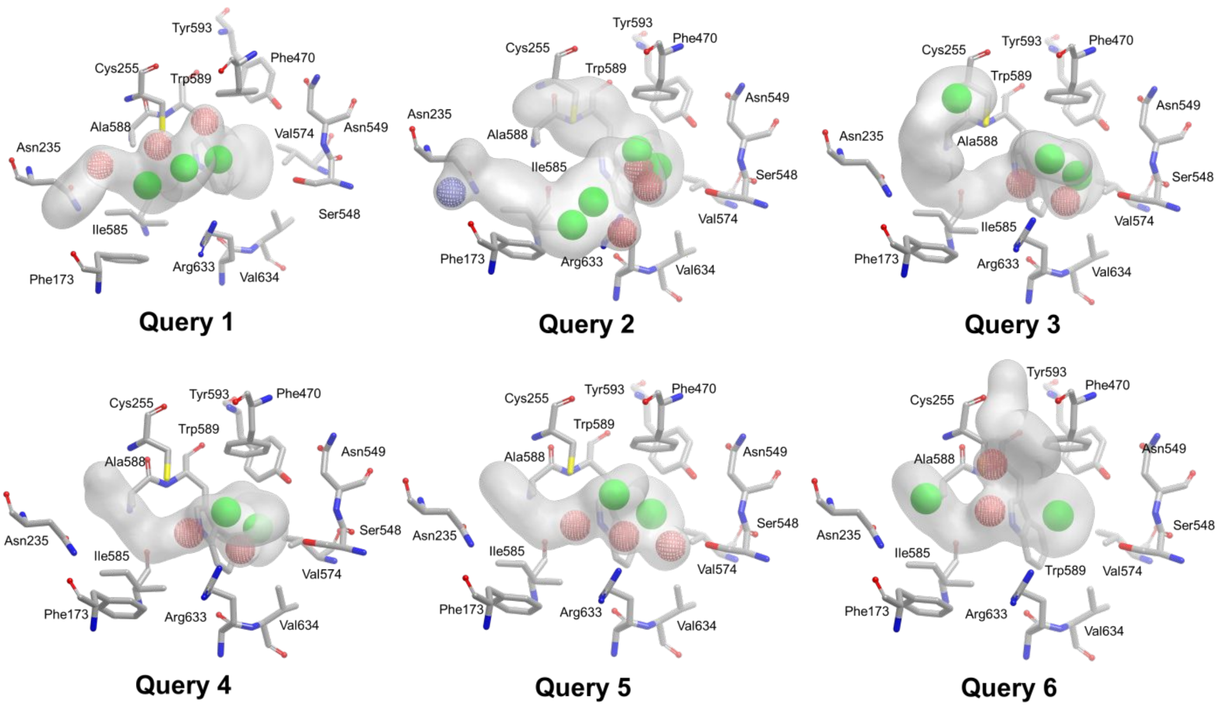

Figure S2. Schematic overview of the shape-based models. The amino acid residues are represented in the shape-based model coordinates to rationalize the color (feature) selection. Green, red, and blue

spheres represent hydrophobic, hydrogen-bond acceptor, and hydrogen-bond donor features, respectively.

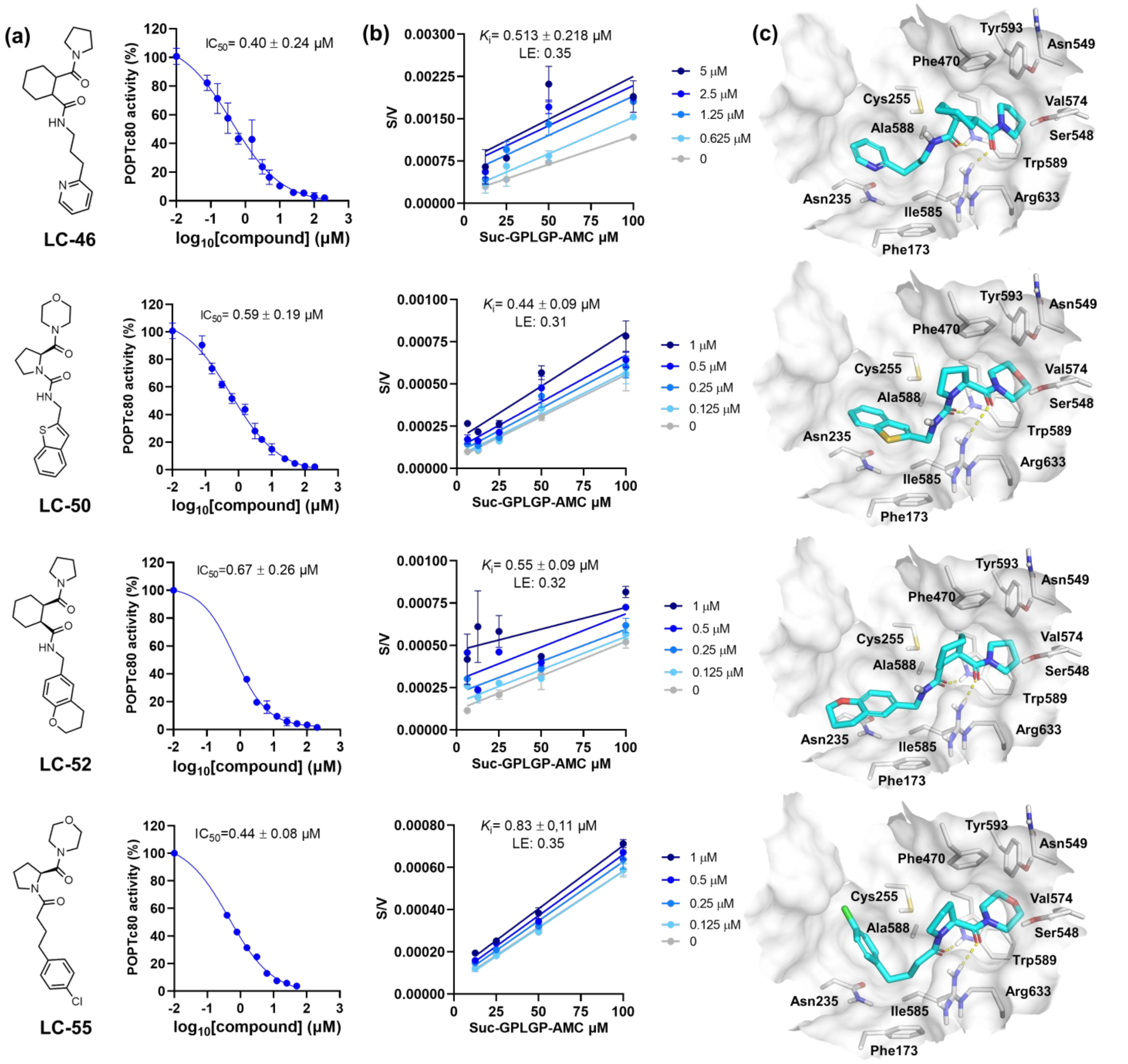

Figure S3. Dose-response curves (**a**), Hanes-Woolf plots (**b**), and predicted binding modes (**c**) of compounds LC-46, LC-50, LC-52, and LC-55 on POPTc80.

6. ^{1}H NMR and LC-MS spectra for test compound

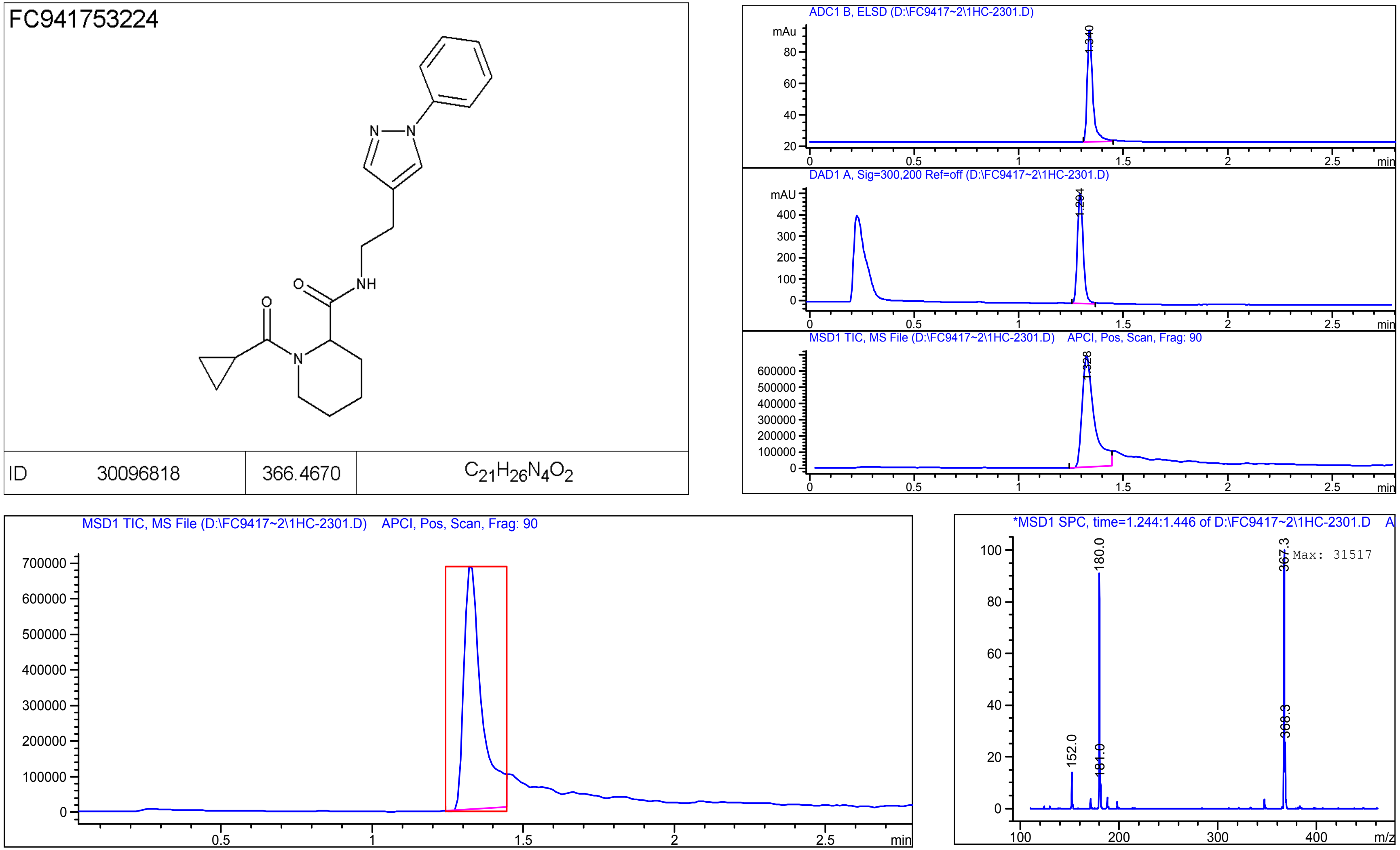

Figure S4. Chromatographic and mass spectra profiles obtained via LC-MS for compound 1-(cyclopropylcarbonyl)-N-[2-(1-phenyl-1H-pyrazol-4-yl)ethyl]piperidine-2-carboxamide (LC-39).

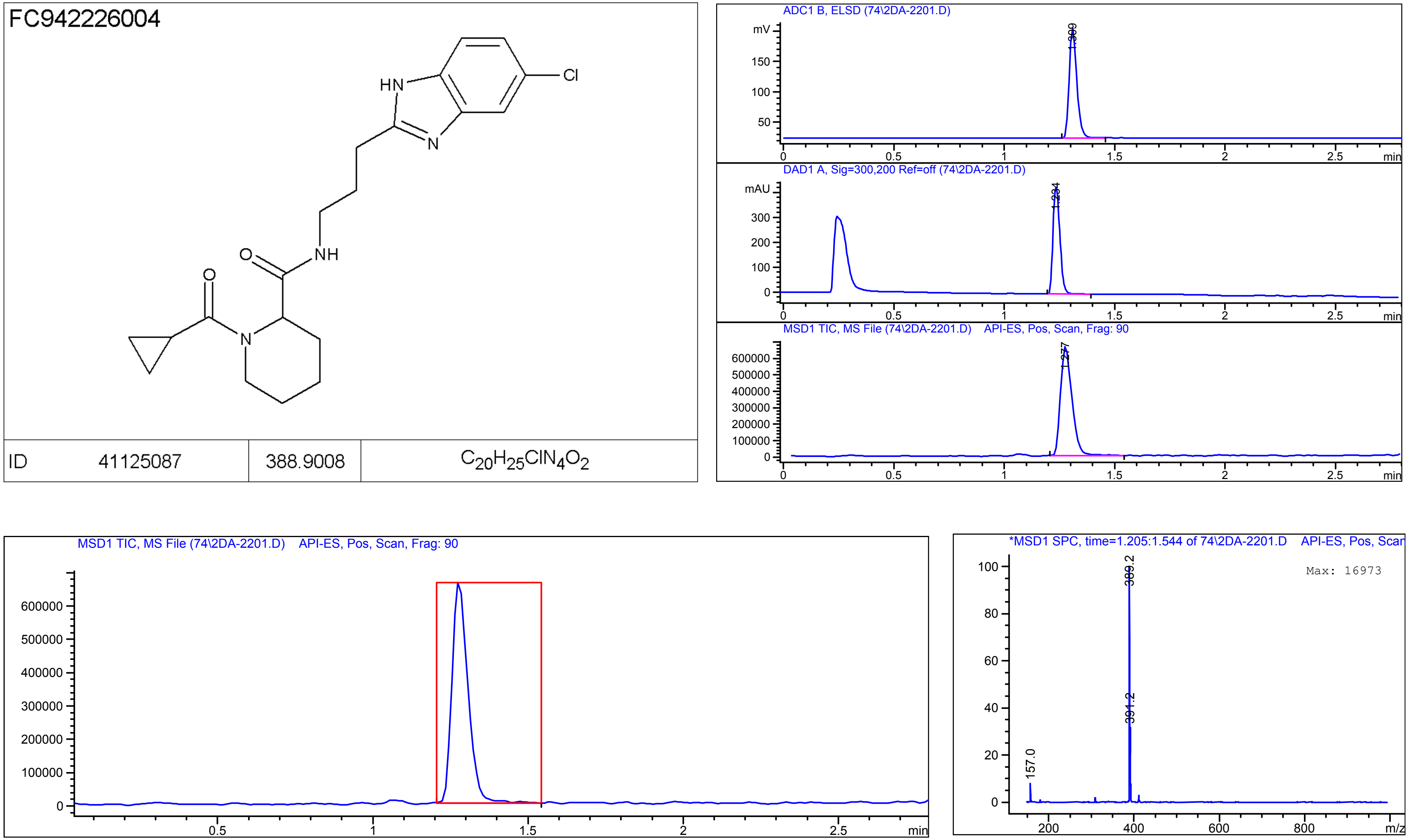

Figure S5. Chromatographic and mass spectra profiles obtained via LC-MS for compound N-[3-(5-chloro-1H-benzimidazol-2-yl)propyl]-1-(cyclopropylcarbonyl)piperidine-2-carboxamide (LC-40).

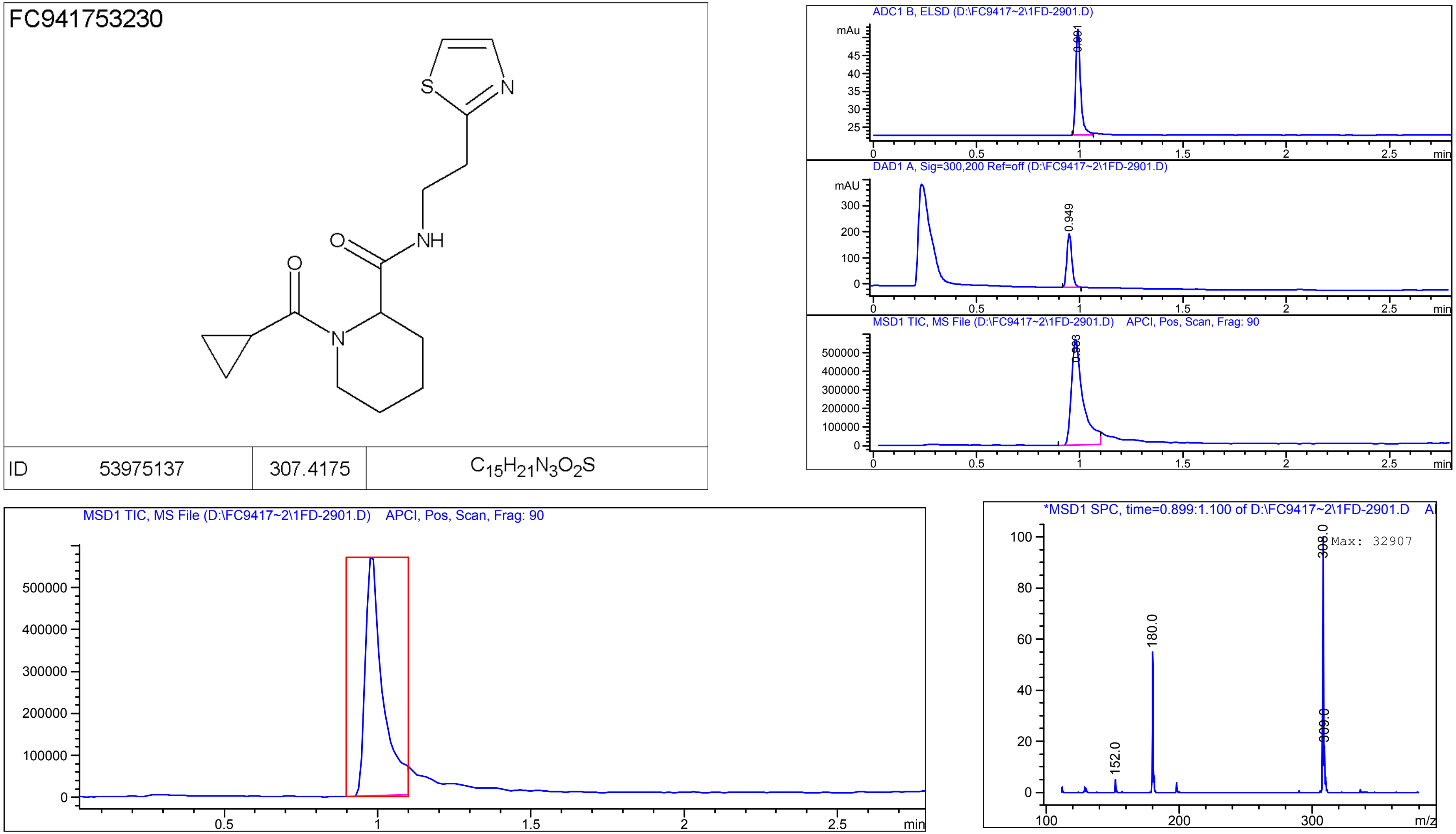

Figure S6. Chromatographic and mass spectra profiles obtained via LC-MS for compound 1-(cyclopropylcarbonyl)-N-[2-(1,3-thiazol-2-yl)ethyl]piperidine-2-carboxamide (LC-41).

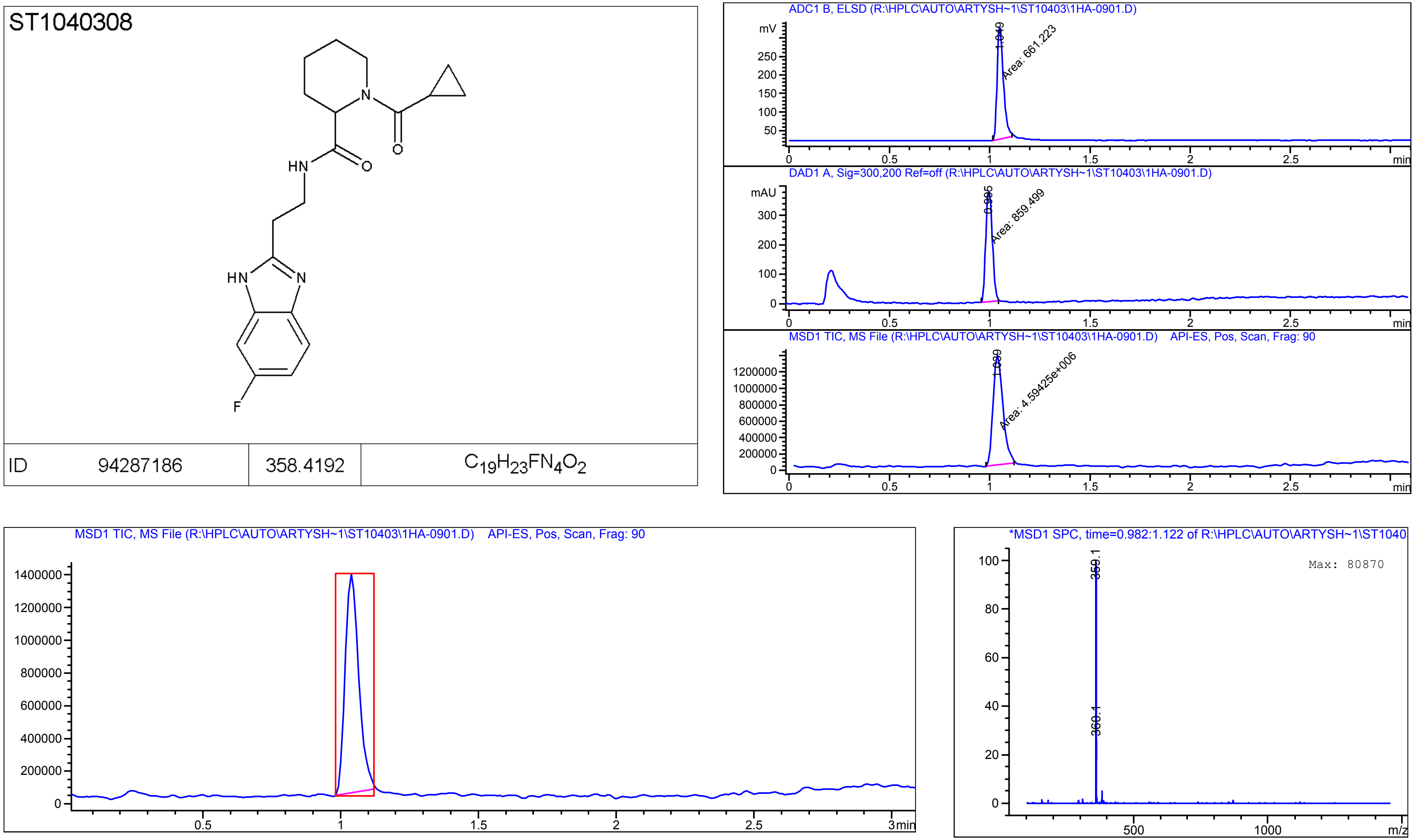

Figure S7. Chromatographic and mass spectra profiles obtained via LC-MS for compound 1-(cyclopropylcarbonyl)-N-[2-(6-fluoro-1H-benzimidazol-2-yl)ethyl]piperidine-2-carboxamide (LC-42).

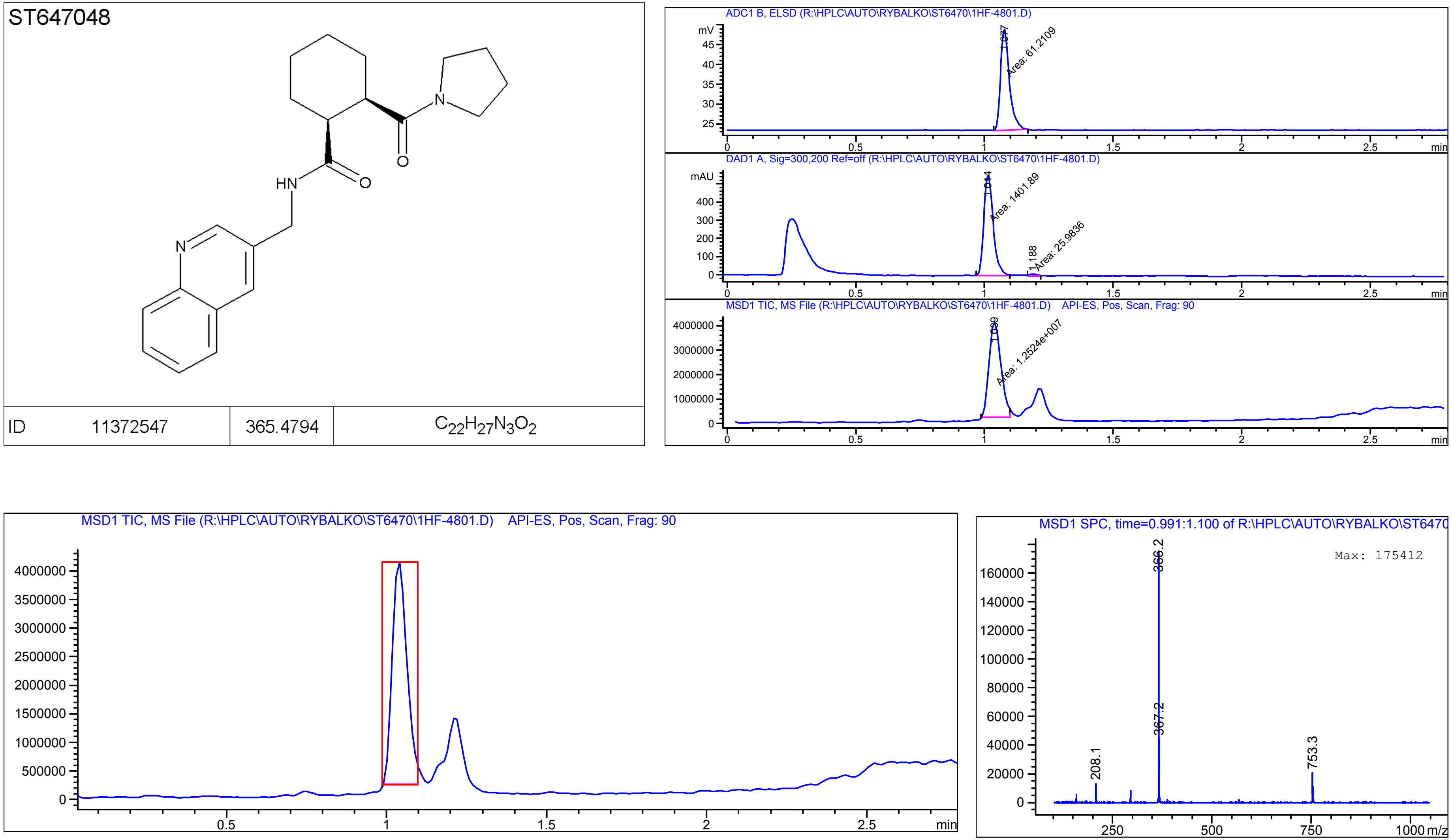

Figure S8. Chromatographic and mass spectra profiles obtained via LC-MS for compound cis-2-(pyrrolidin-1-ylcarbonyl)-N-(quinolin-3-ylmethyl)cyclohexanecarboxamide (LC-43).

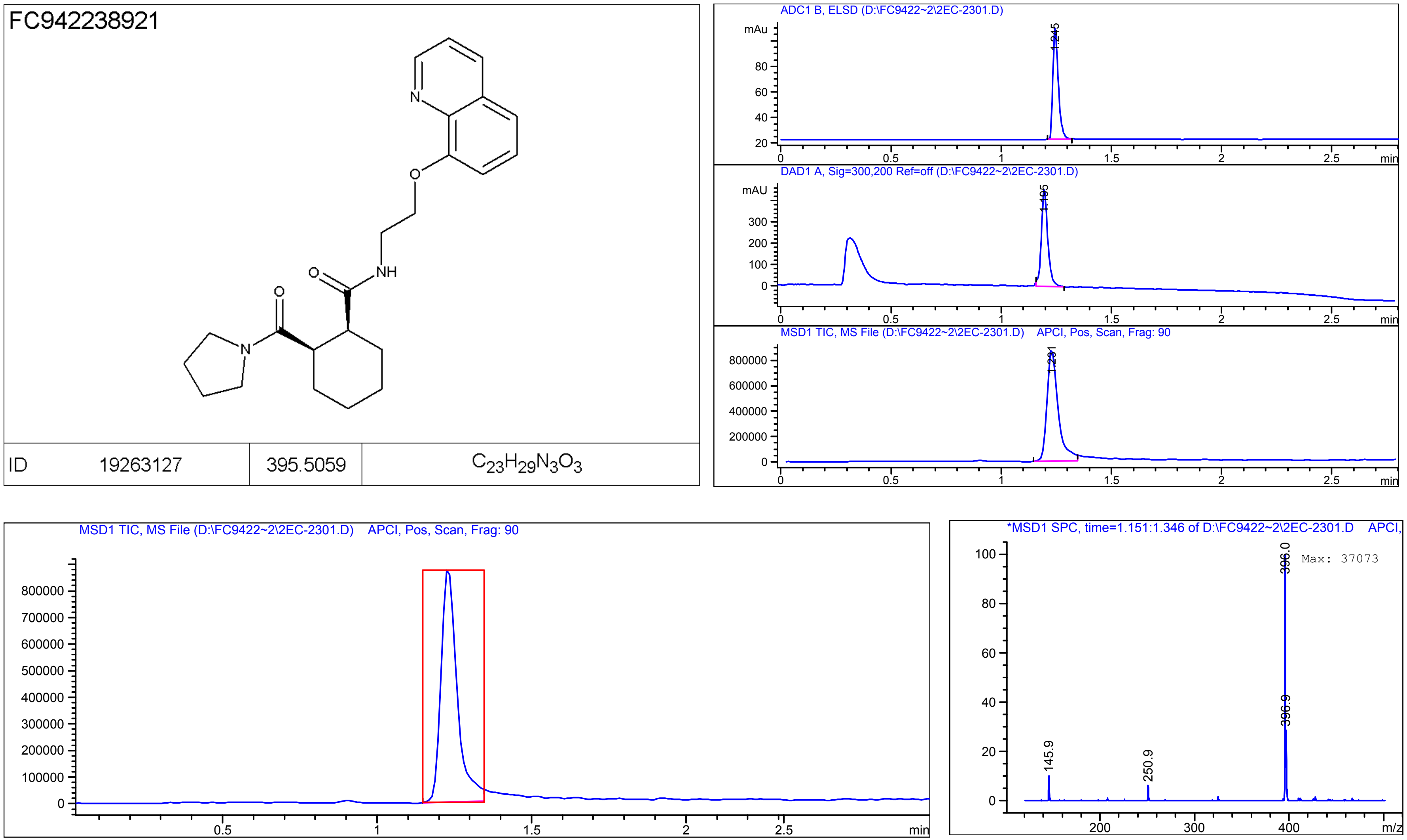

Figure S9. Chromatographic and mass spectra profiles obtained via LC-MS for compound rac-(1S,2R)-2-(pyrrolidin-1-ylcarbonyl)-N-[2-(quinolin-8-yloxy)ethyl]cyclohexanecarboxamide (LC-44).

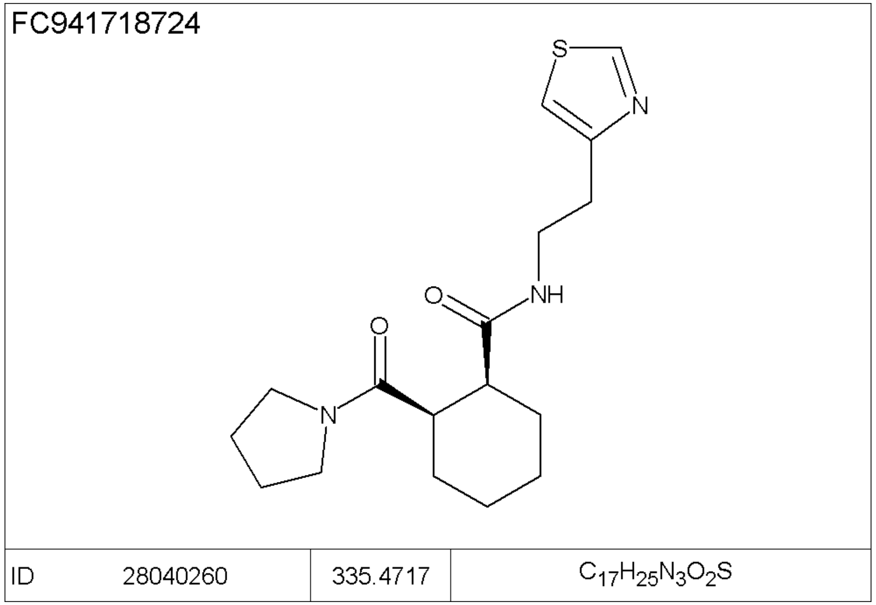

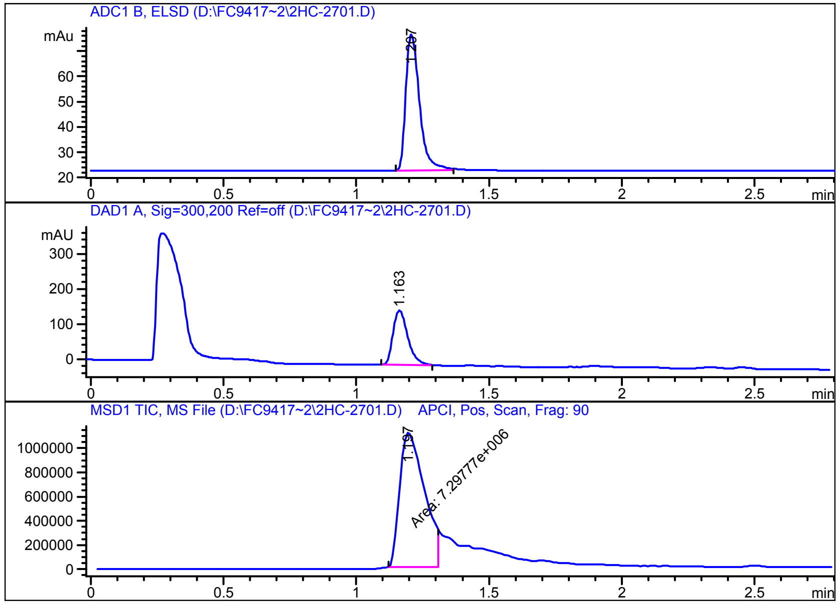

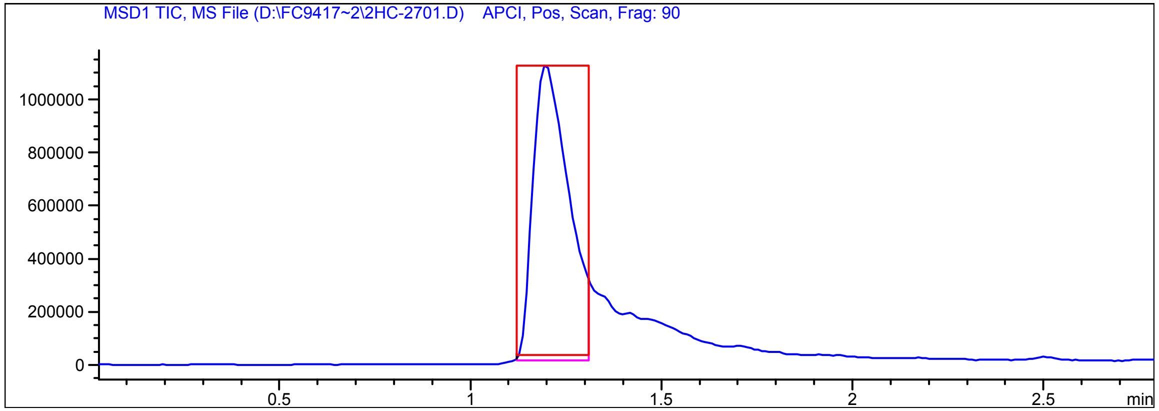

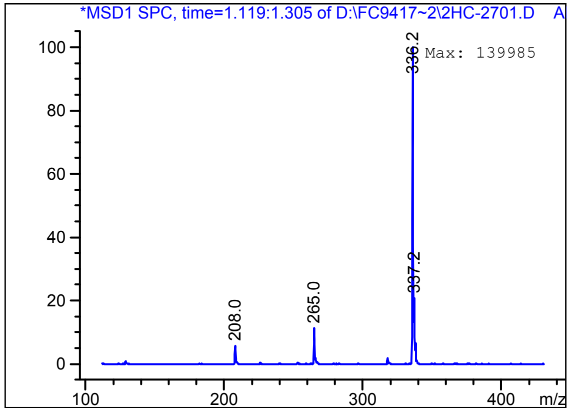

Figure S10. Chromatographic and mass spectra profiles obtained via LC-MS for compound (1S*,2R*)-2-(pyrrolidin-1-ylcarbonyl)-N-[2-(1,3-thiazol-4-yl)ethyl]cyclohexanecarboxamide (LC-45).

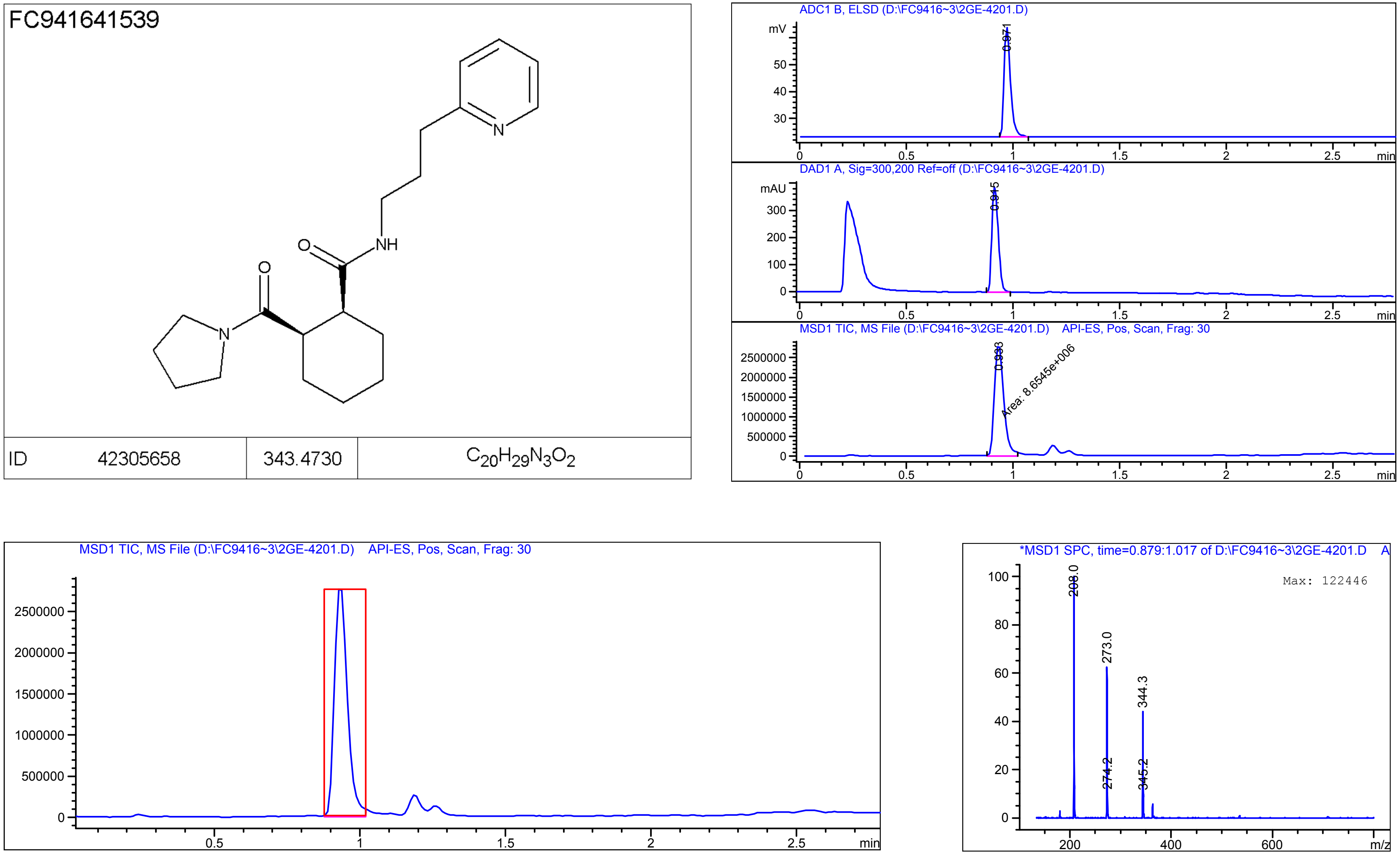

Figure S11. Chromatographic and mass spectra profiles obtained via LC-MS for compound (1S*,2R*)-N-(3-pyridin-2-ylpropyl)-2-(pyrrolidin-1-ylcarbonyl)cyclohexanecarboxamide (LC-46).

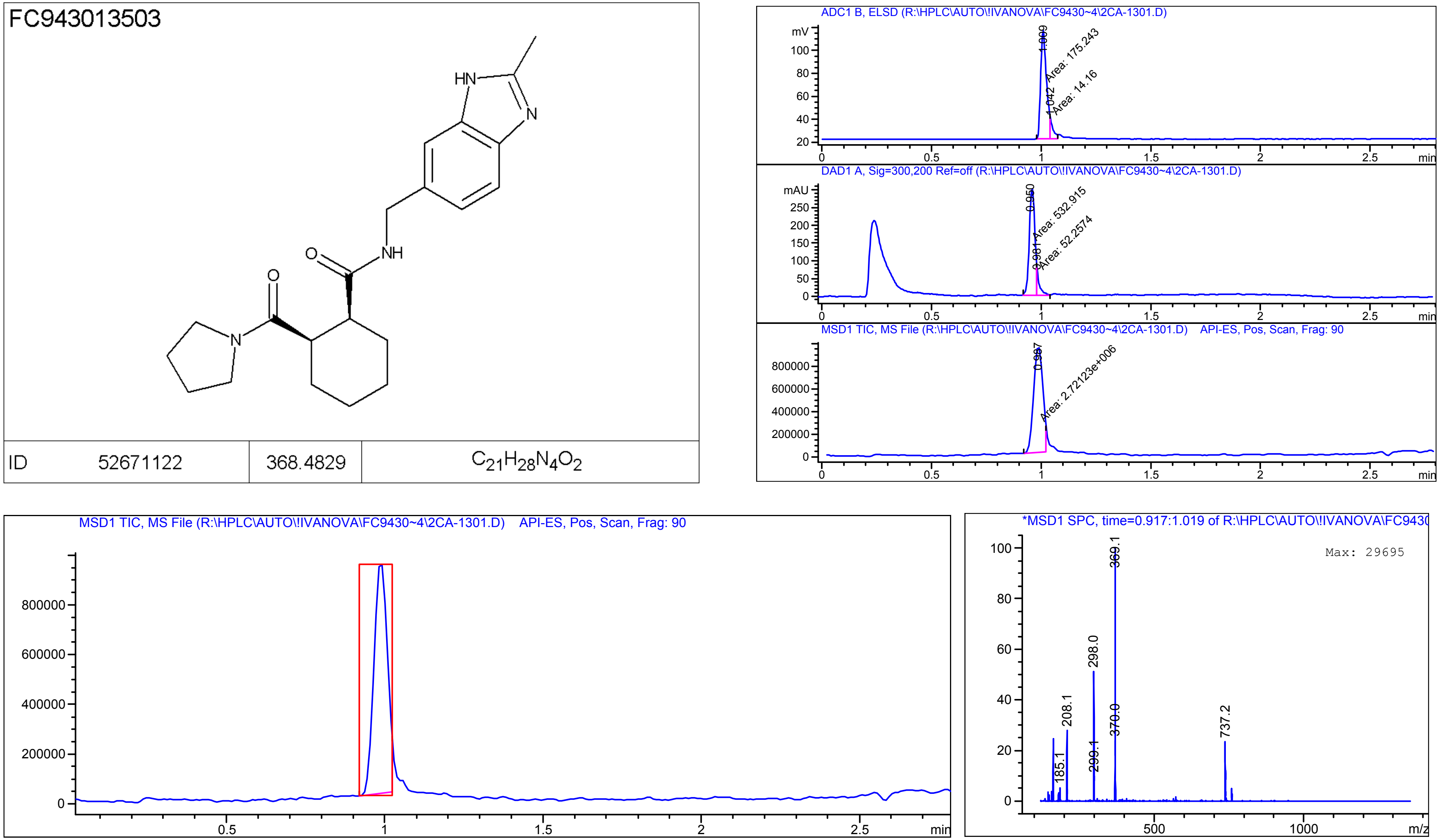

Figure S12. Chromatographic and mass spectra profiles obtained via LC-MS for compound (1S*,2R*)-N-[(2-methyl-1H-benzimidazol-6-yl)methyl]-2-(pyrrolidin-1-ylcarbonyl)cyclohexanecarboxamide (LC-47).

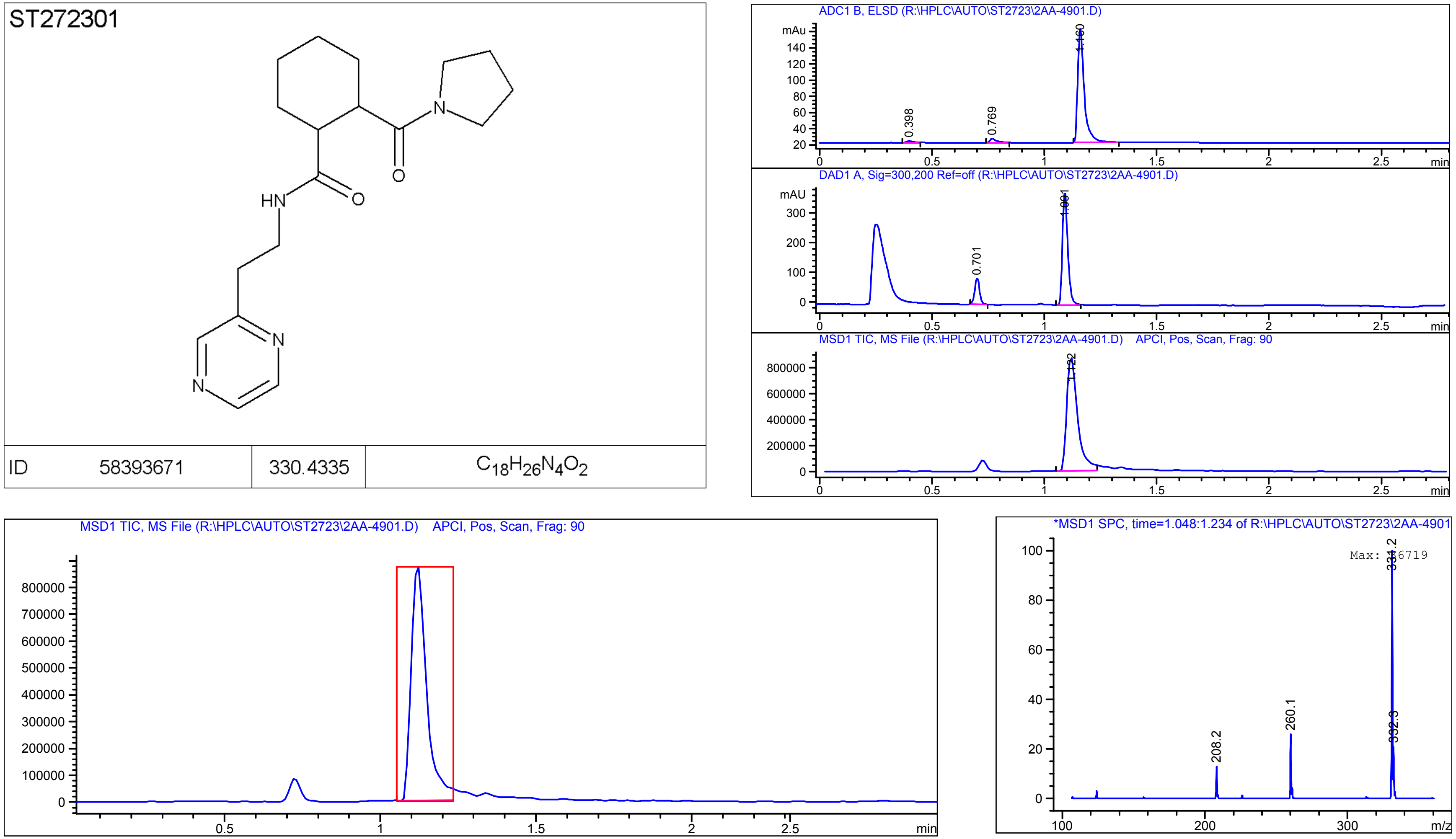

Figure S13. Chromatographic and mass spectra profiles obtained via LC-MS for compound N-[2-(2-pyrazinyl)ethyl]-2-(1-pyrrolidinylcarbonyl)cyclohexanecarboxamide (LC-48).

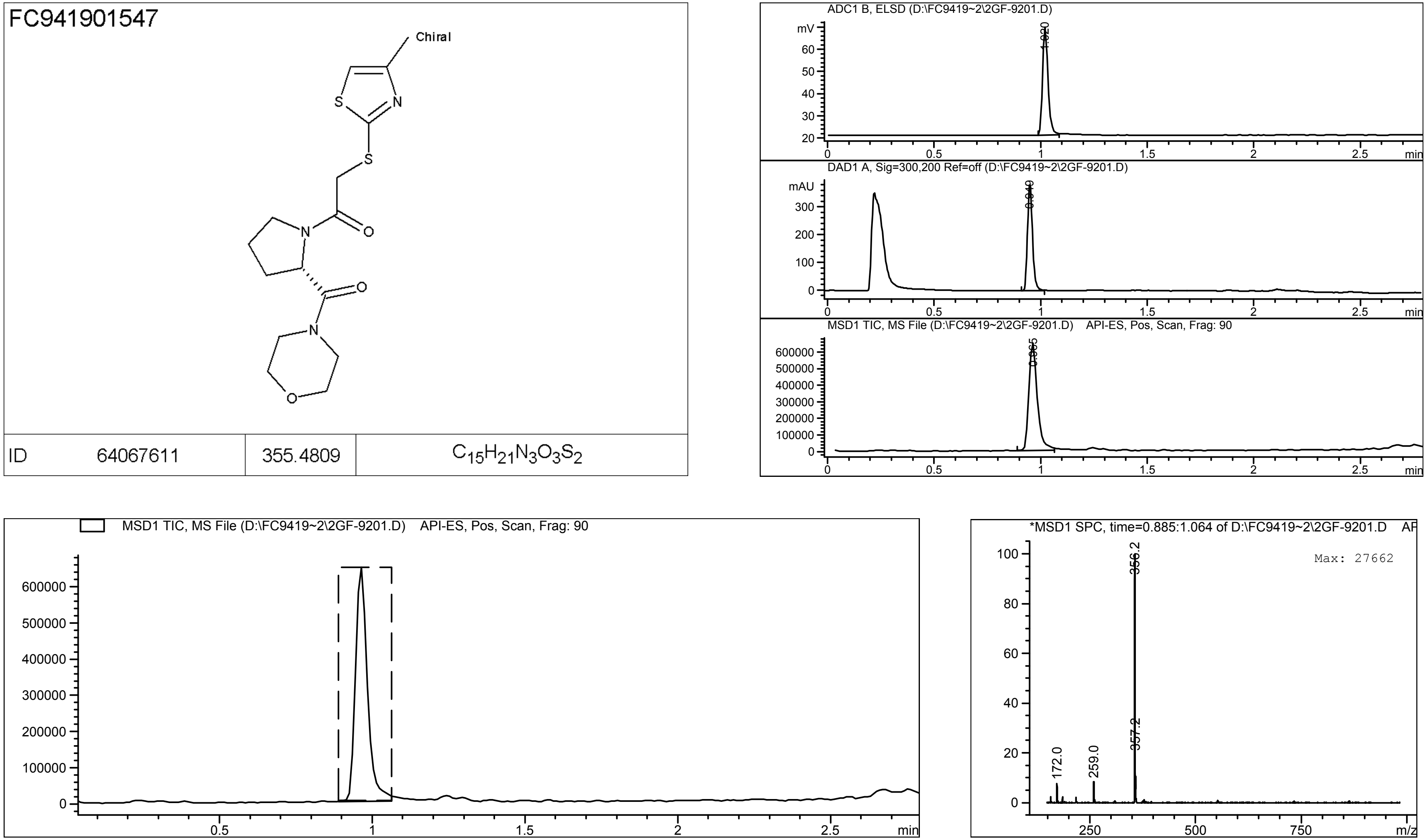

Figure S14. Chromatographic and mass spectra profiles obtained via LC-MS for compound 4-(1-{[(4-methyl-1,3-thiazol-2-yl)thio]acetyl}-L-prolyl)morpholine (LC-49).

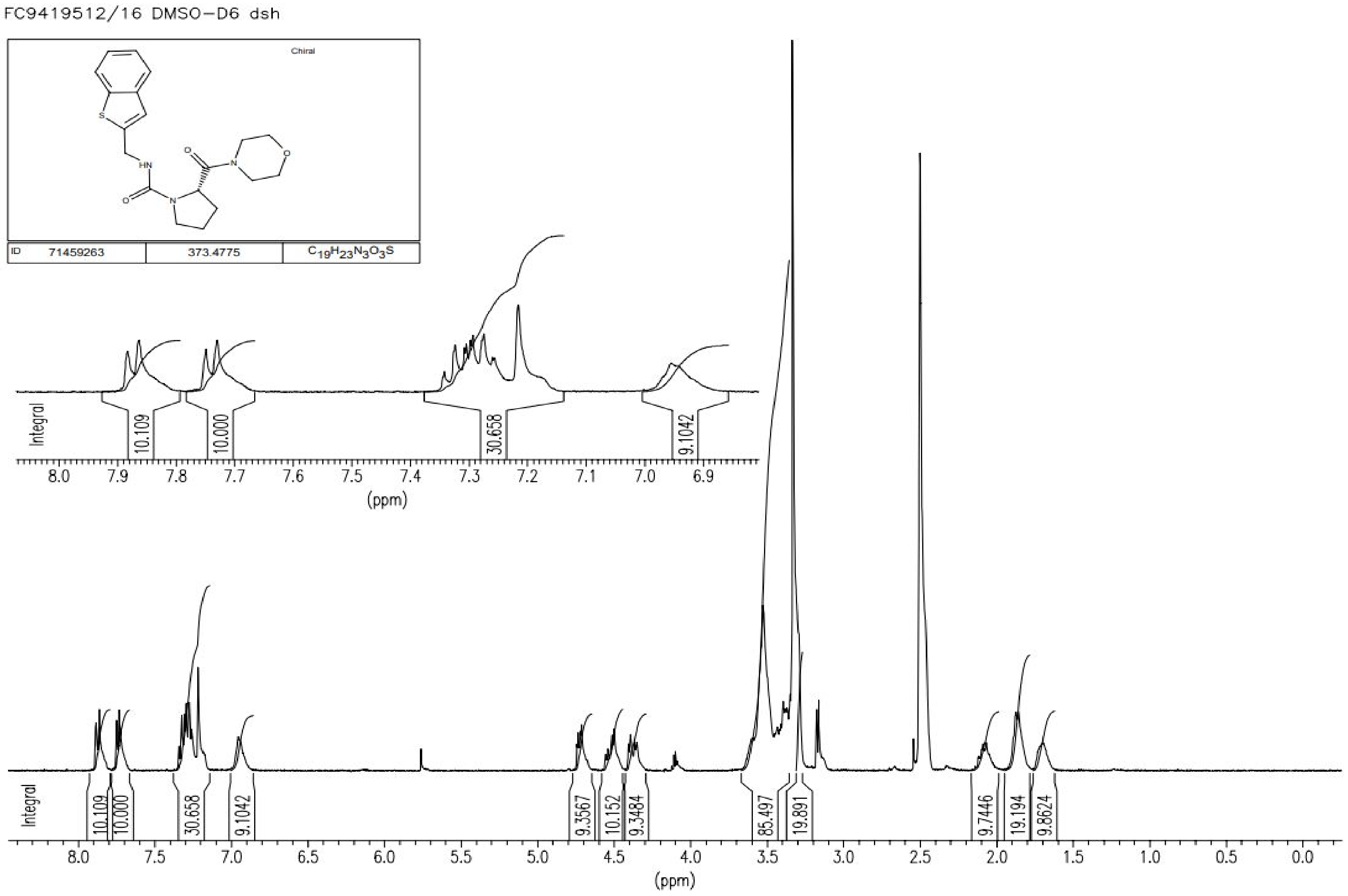

Figure S15. ^{1}H NMR spectrum of compound (2S)-N-(1-benzothien-2-ylmethyl)-2-(morpholin-4-ylcarbonyl)pyrrolidine-1-carboxamide (LC-50).

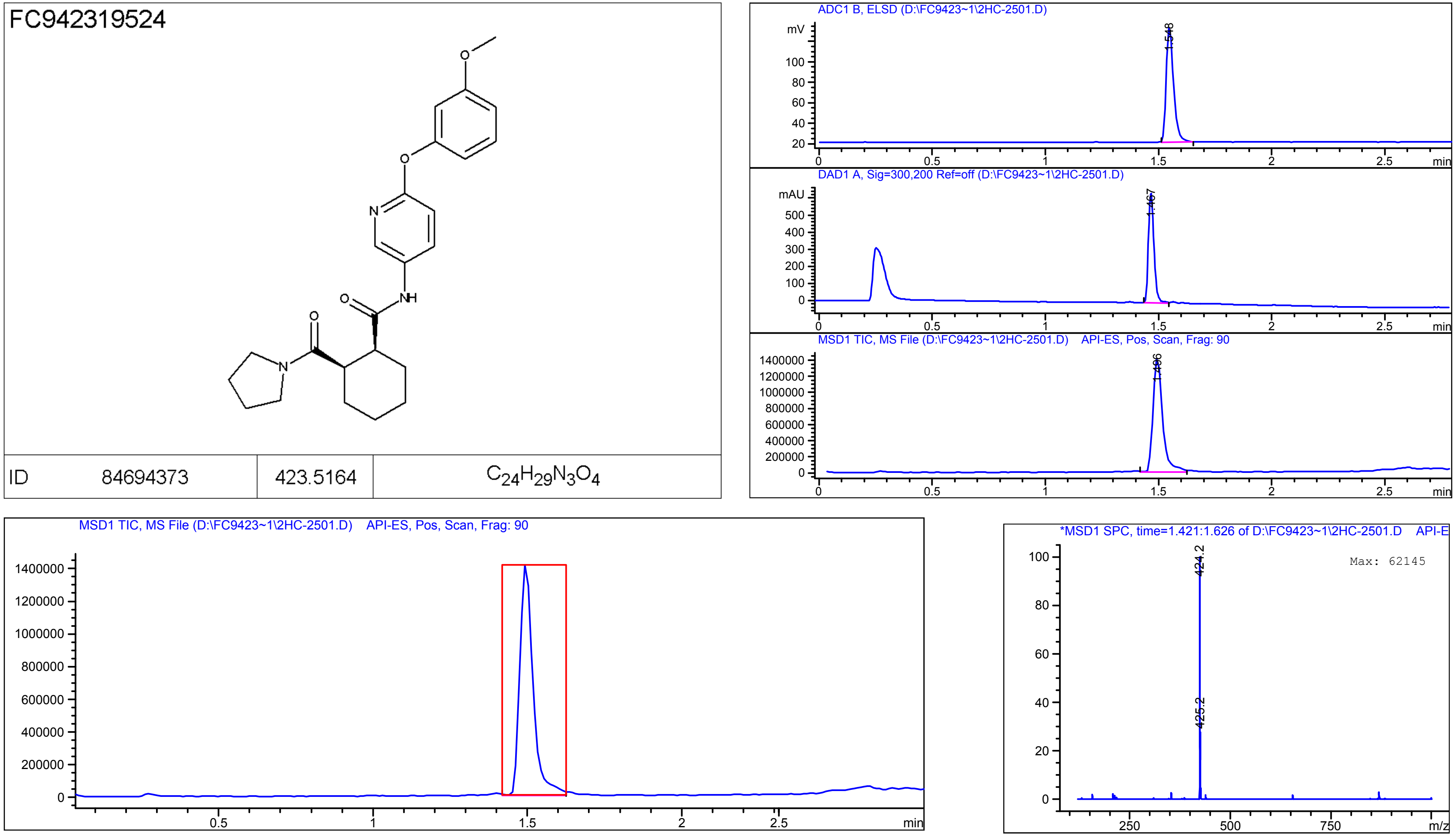

Figure S16. Chromatographic and mass spectra profiles obtained via LC-MS for compound rac-(1S,2R)-N-[6-(3-methoxyphenoxy)pyridin-3-yl]-2-(pyrrolidin-1-ylcarbonyl)cyclohexanecarboxamide (LC-51).

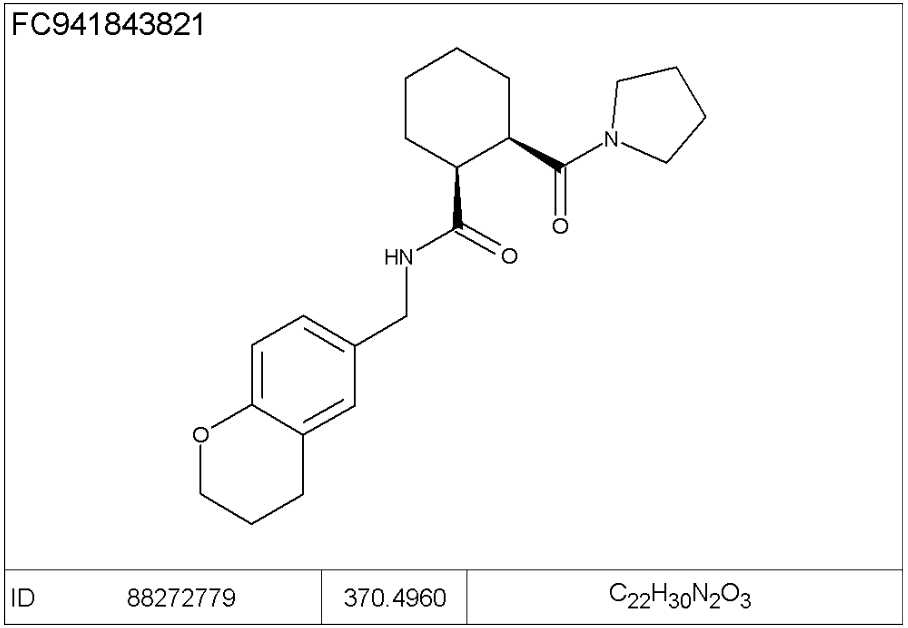

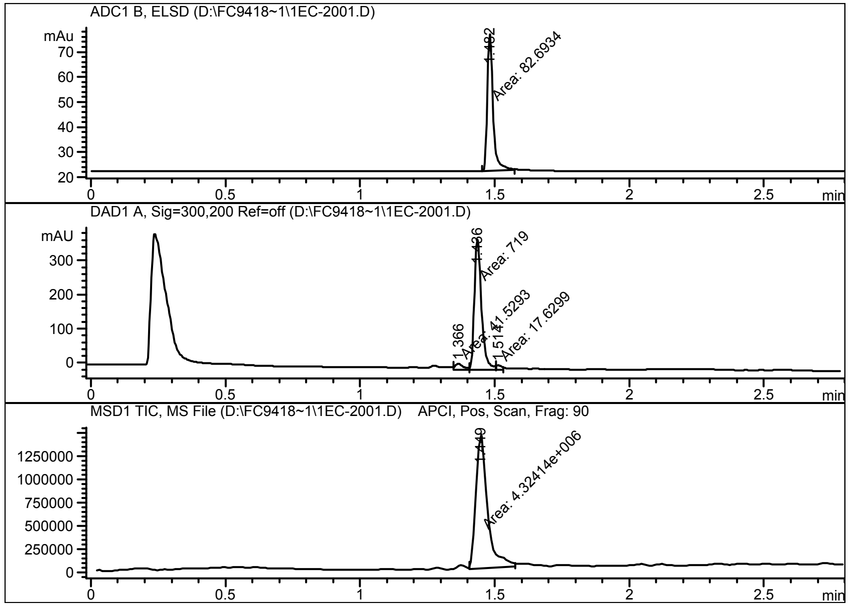

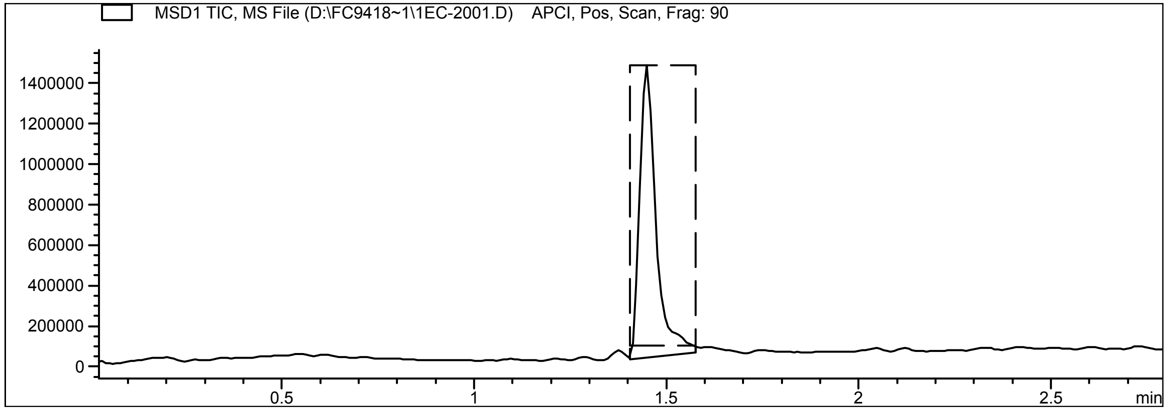

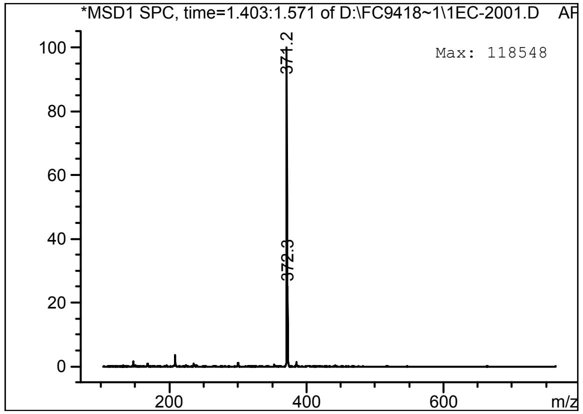

Figure S17. Chromatographic and mass spectra profiles obtained via LC-MS for compound cis-N-(3,4-dihydro-2H-chromen-6-ylmethyl)-2-(pyrrolidin-1-ylcarbonyl)cyclohexanecarboxamide (LC-52).

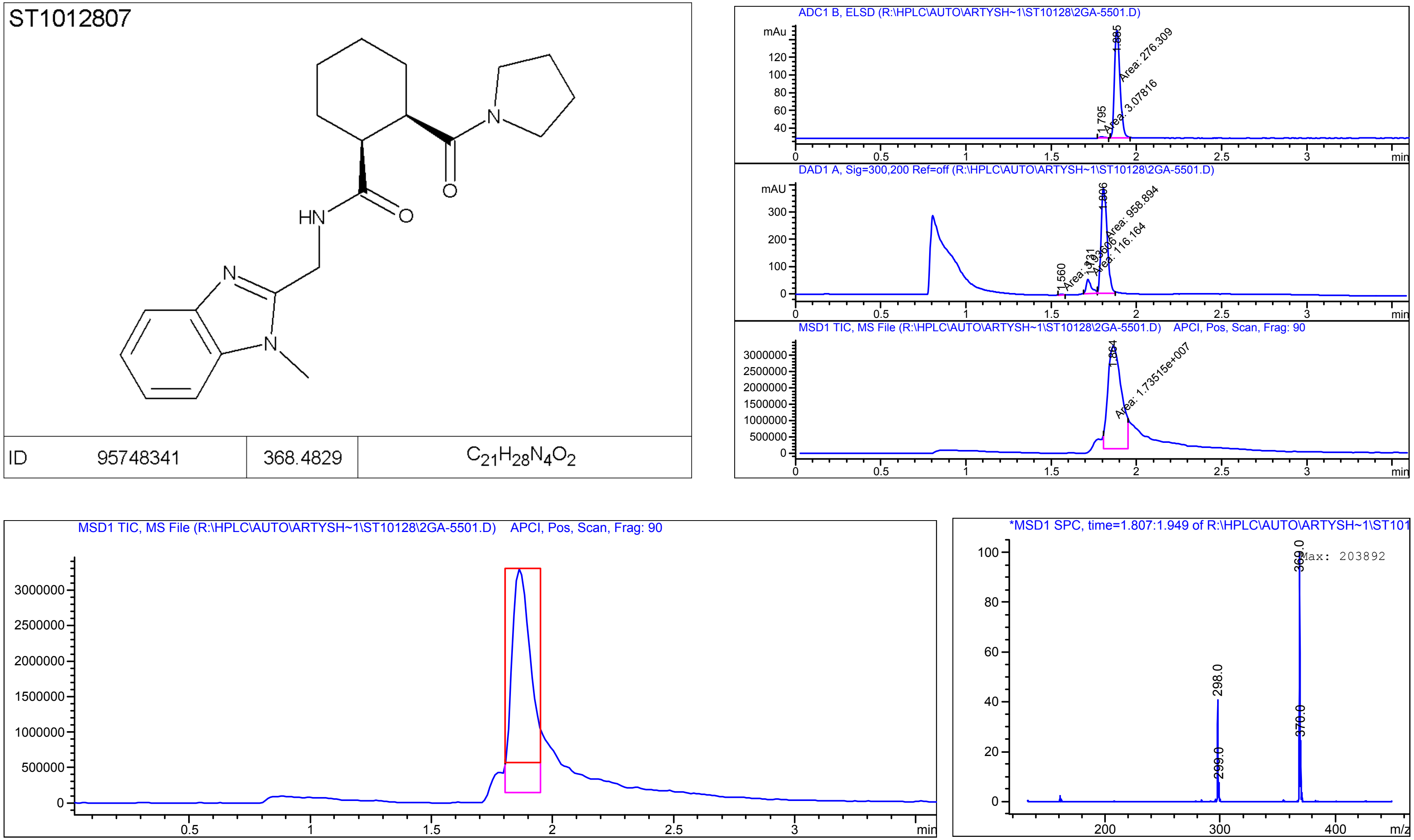

Figure S18. Chromatographic and mass spectra profiles obtained via LC-MS for compound cis-N-[(1-methyl-1H-benzimidazol-2-yl)methyl]-2-(pyrrolidin-1-ylcarbonyl)cyclohexanecarboxamide (LC-53).

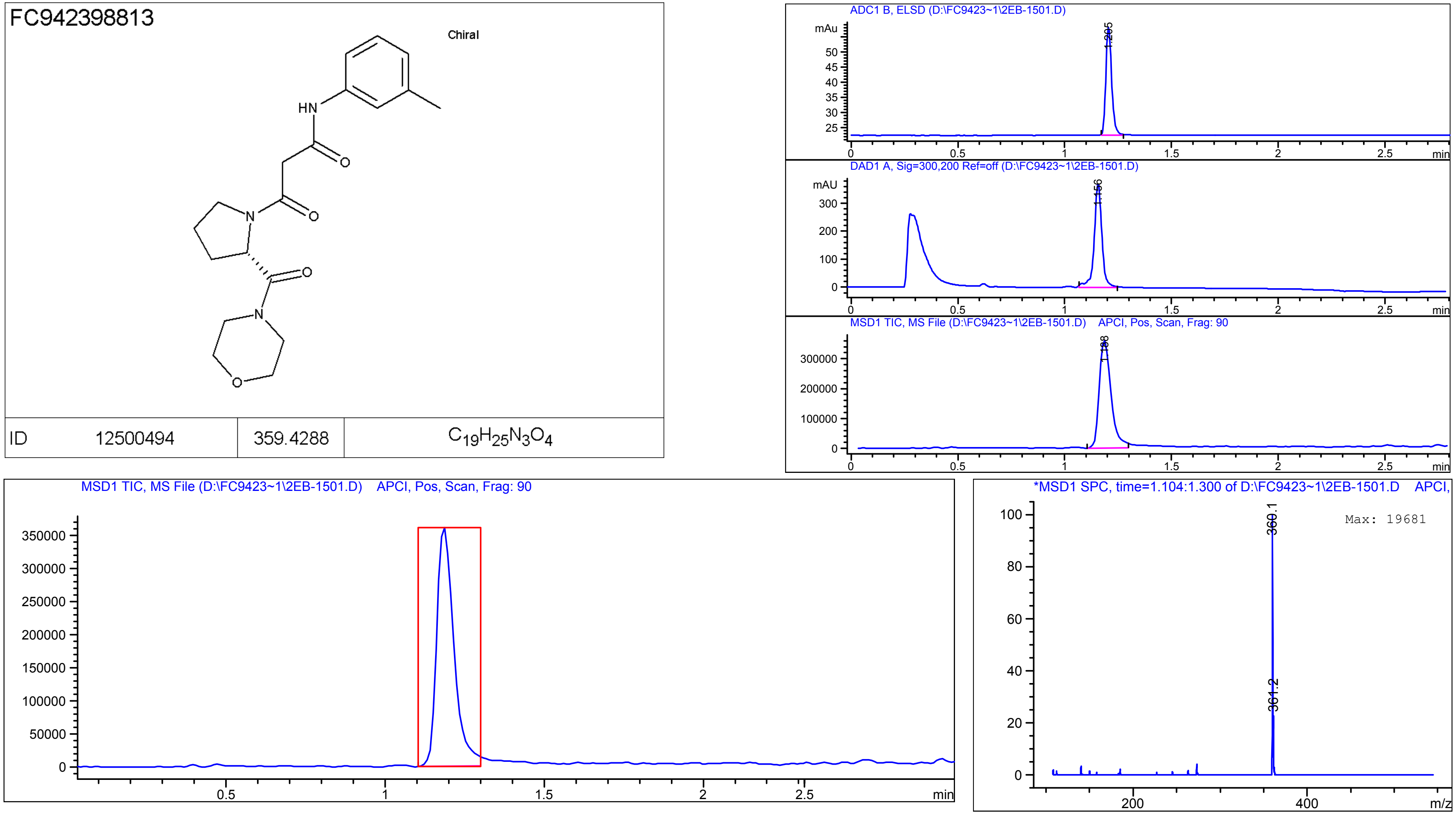

Figure S19. Chromatographic and mass spectra profiles obtained via LC-MS for compound N-(3-methylphenyl)-3-[(2S)-2-(morpholin-4-ylcarbonyl)pyrrolidin-1-yl]-3-oxopropanamide (LC-54).

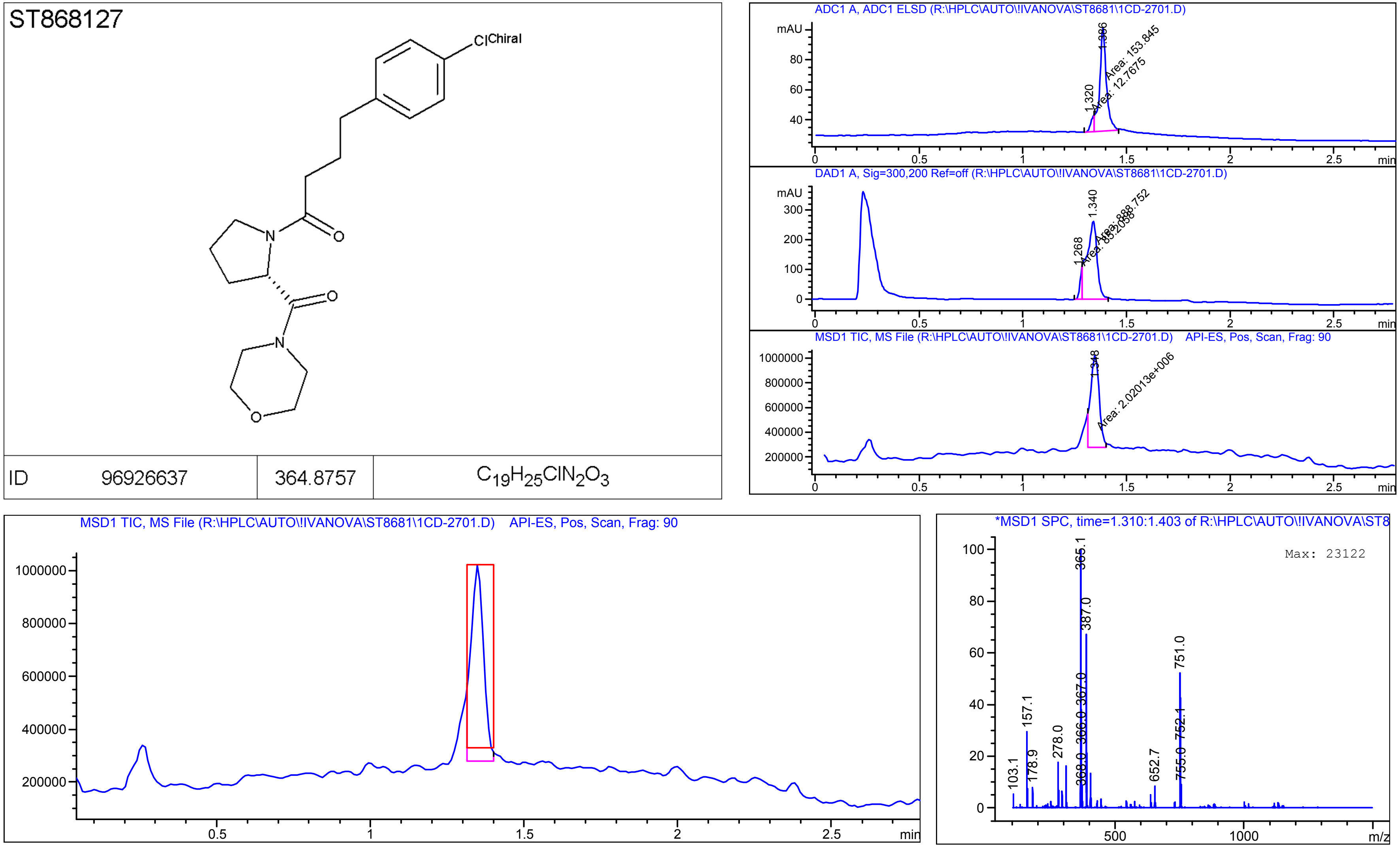

Figure S20. Chromatographic and mass spectra profiles obtained via LC-MS for compound 4-{1-[4-(4-chlorophenyl)butanoyl]-L-prolyl}morpholine (LC-55).

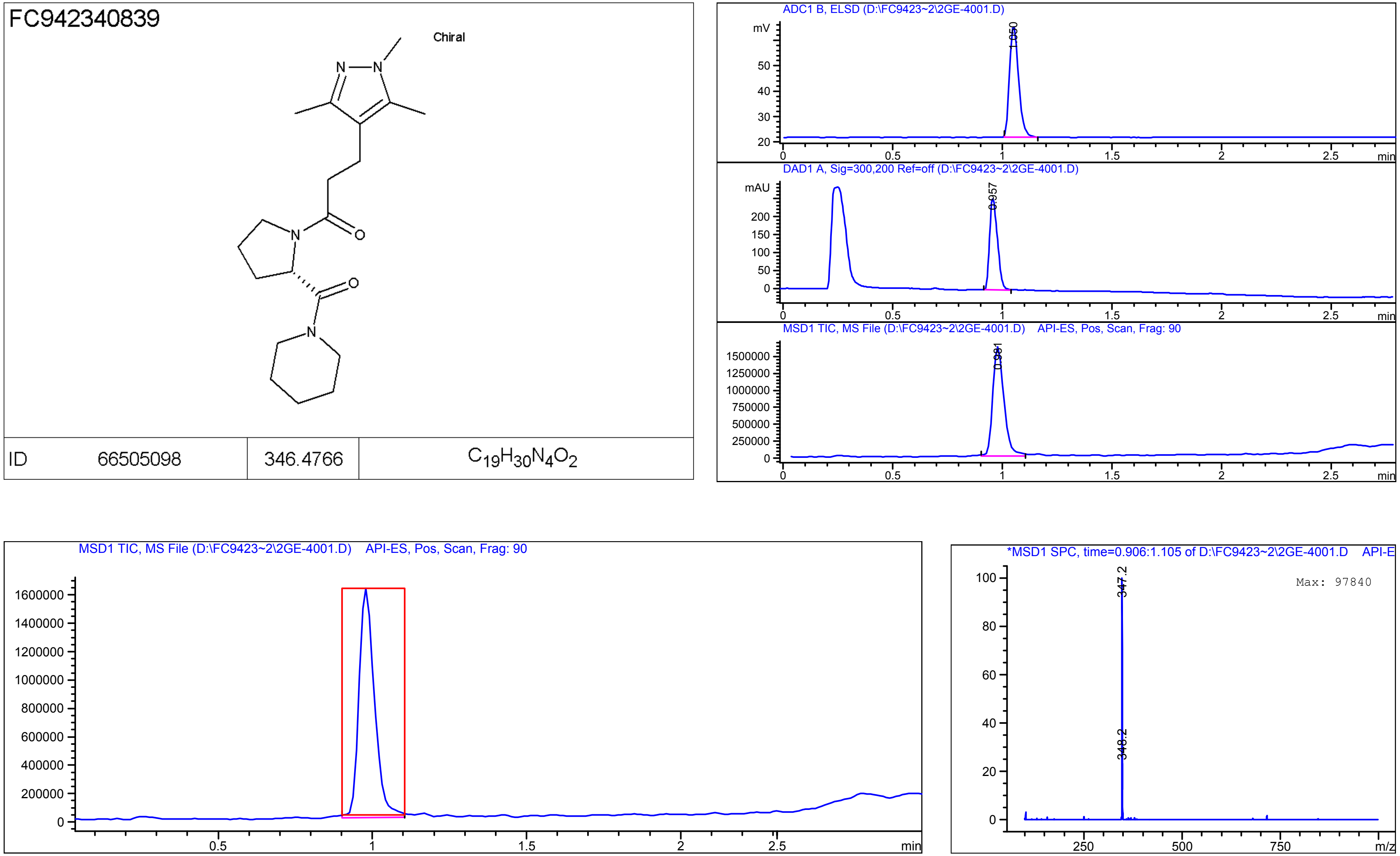

Figure S21. Chromatographic and mass spectra profiles obtained via LC-MS for compound 1-({(2S)-1-[3-(1,3,5-trimethyl-1H-pyrazol-4-yl)propanoyl]pyrrolidin-2-yl}carbonyl)piperidine (LC-56).

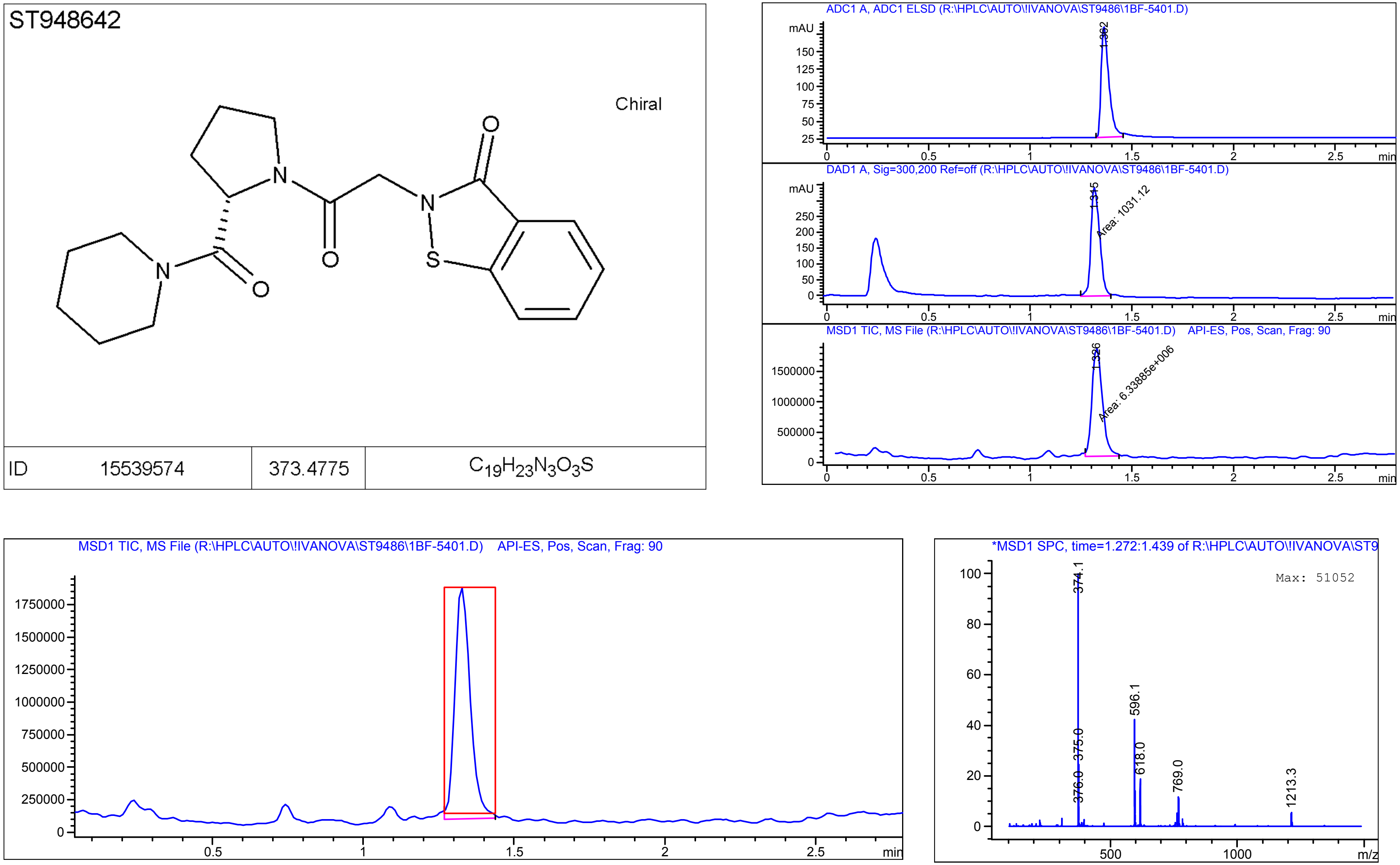

Figure S22. Chromatographic and mass spectra profiles obtained via LC-MS for compound 2-{2-oxo-2-[(2S)-2-(piperidin-1-ylcarbonyl)pyrrolidin-1-yl]ethyl}-1,2-benzisothiazol-3(2H)-one (LC-57).

References

1. Xiong G, Wu Z, Yi J, Fu L, Yang Z, Hsieh C, Yin M, Zeng X, Wu C, Lu A, Chen X, Hou T, Cao D (2021) ADMETlab 2.0: an integrated online platform for accurate and comprehensive predictions of ADMET properties. Nucleic Acids Research 49:W5–W14. https://doi.org/10.1093/nar/gkab255

2. Bal G, Van der Veken P, Antonov D, Lambeir A-M, Grellier P, Croft SL, Augustyns K, Haemers A (2003) Prolylisoxazoles: potent inhibitors of prolyloligopeptidase with antitrypanosomal activity. Bioorganic & Medicinal Chemistry Letters 13:2875–2878. https://doi.org/10.1016/S0960-894X(03)00579-1

3. de Almeida H, Leroux V, Motta FN, Grellier P, Maigret B, Santana JM, Bastos IMD (2016) Identification of novel Trypanosoma cruzi prolyl oligopeptidase inhibitors by structure-based virtual screening. Journal of Computer-Aided Molecular Design 30:1165–1174. https://doi.org/10.1007/s10822-016-9985-1

4. VENDEVILLE S, BUISINE E, WILLIARD X, SCHREVEL J, GRELLIER P, SANTANA J, SERGHERAERT C (1999) Identification of Inhibitors of an 80kDa Protease from Trypanosoma cruzi through the Screening of a Combinatorial Peptide Library. CHEMICAL & PHARMACEUTICAL BULLETIN 47:194–198. https://doi.org/10.1248/cpb.47.194

5. Joyeau R, Maoulida C, Guillet C, Frappier F, Teixeira ARL, Schrével J, Santana J, Grellier P (2000) Synthesis and activity of pyrrolidinyl- and thiazolidinyl-dipeptide derivatives as inhibitors of the Tc80 prolyl oligopeptidase from Trypanosoma cruzi. European Journal of Medicinal Chemistry 35:257–266. https://doi.org/10.1016/S0223-5234(00)00118-5

6. Grellier P, Vendeville S, Joyeau R, Bastos IMD, Drobecq H, Frappier F, Teixeira ARL, Schrével J, Davioud-Charvet E, Sergheraert C, Santana JM (2001) Trypanosoma cruzi Prolyl Oligopeptidase Tc80 Is Involved in Nonphagocytic Mammalian Cell Invasion by Trypomastigotes. Journal of Biological Chemistry 276:47078–47086. https://doi.org/10.1074/jbc.M106017200

7. Elhalem E, Bailey BN, Docampo R, Ujváry I, Szajnman SH, Rodriguez JB (2002) Design, Synthesis, and Biological Evaluation of Aryloxyethyl Thiocyanate Derivatives against Trypanosoma cruzi. J Med Chem 45:3984–3999. https://doi.org/10.1021/jm0201518

8. Vendeville S, Bourel L, Davioud-Charvet E, Grellier P, Deprez B, Sergheraert C (1999) Automated parallel synthesis of a tetrahydroisoquinolin-based library: Potential prolyl endopeptidase inhibitors. Bioorganic & Medicinal Chemistry Letters 9:437–442. https://doi.org/10.1016/S0960-894X(99)00003-7

9. Russell S, Rahmani R, Jones AJ, Newson HL, Neilde K, Cotillo I, Rahmani Khajouei M, Ferrins L, Qureishi S, Nguyen N, Martinez-Martinez MS, Weaver DF, Kaiser M, Riley J, Thomas J, De Rycker M, Read KD, Flematti GR, Ryan E, Tanghe S, Rodriguez A, Charman SA, Kessler A, Avery VM, Baell JB, Piggott MJ (2016) Hit-to-Lead Optimization of a Novel Class of Potent, Broad-Spectrum Trypanosomacides. J Med Chem 59:9686–9720. https://doi.org/10.1021/acs.jmedchem.6b00442

10. Peña I, Pilar Manzano M, Cantizani J, Kessler A, Alonso-Padilla J, Bardera AI, Alvarez E, Colmenarejo G, Cotillo I, Roquero I, de Dios-Anton F, Barroso V, Rodriguez A, Gray DW, Navarro M, Kumar V, Sherstnev A, Drewry DH, Brown JR, Fiandor JM, Julio Martin J (2015)

New Compound Sets Identified from High Throughput Phenotypic Screening Against Three Kinetoplastid Parasites: An Open Resource. Sci Rep 5:8771. https://doi.org/10.1038/srep08771

11. Ellis-Guardiola K, Rui H, Beckner RL, Srivastava P, Sukumar N, Roux B, Lewis JC (2019) Crystal Structure and Conformational Dynamics of Pyrococcus furiosus Prolyl Oligopeptidase. Biochemistry 58:1616–1626. https://doi.org/10.1021/acs.biochem.9b00031
